# Epigenetically modulated FOXM1 suppresses dendritic cell maturation in pancreatic cancer and colon cancer

**DOI:** 10.1002/1878-0261.12443

**Published:** 2019-02-15

**Authors:** Zhongshi Zhou, Hongdan Chen, Rui Xie, Hongjie Wang, Senlin Li, Qianqian Xu, Na Xu, Qi Cheng, Ying Qian, Rongrong Huang, Zekun Shao, Ming Xiang

**Affiliations:** ^1^ Department of Pharmacology School of Pharmacy Tongji Medical College Huazhong University of Science and Technology Wuhan People's Republic of China; ^2^ Section of Neurobiology Torrey Pines Institute for Molecular Studies Port St Lucie FL USA

**Keywords:** colon cancer, dendritic cell, epigenetic modification, FOXM1, H3K79me2, pancreatic cancer

## Abstract

Forkhead box transcription factor M1 (FOXM1) is a proliferation‐associated transcription factor involved in tumorigenesis through transcriptional regulation of its target genes in various cells, including dendritic cells (DCs). Although previous work has shown that FOXM1 enhances DC maturation in response to house dust mite allergens, it is not known whether FOXM1 affects DC maturation in the context of tumor‐specific immunity. In this study, we examined the central role of FOXM1 in regulating bone marrow‐derived dendritic cell (BMDC) maturation phenotypes and function in pancreatic cancer and colon cancer. FOXM1 retarded maturation phenotypes of BMDCs, inhibited promotion of T‐cell proliferation, and decreased interleukin‐12 (IL‐12) p70 in tumor‐bearing mice (TBM). Notably, FOXM1 expression was epigenetically regulated by dimethylation on H3 lysine 79 (H3K79me2), a modification present in both tumor cells and BMDCs. Increased H3K79me2 enrichment was observed at the FOXM1 promoter in both BMDCs from TBM, and in BMDCs from wild‐type mice cultured with tumor‐conditioned medium that mimics the tumor microenvironment (TME). Furthermore, inhibition of the H3K79 methyltransferase DOT1L not only decreased enrichment of H3K79me2, but also downregulated expression of FOXM1 and partially reversed its immunosuppressive effects on BMDCs. Furthermore, we found that FOXM1 upregulated transcription of Wnt family number 5A (Wnt5a) in BMDCs *in vitro*; we also observed that exogenous Wnt5a expression abrogated BMDC maturation phenotypes by inhibiting FOXM1 and H3K79me2 modification. Therefore, our results reveal that upregulation of FOXM1 by H3K79me2 in pancreatic cancer and colon cancer significantly inhibits maturation phenotypes and function of BMDCs through the Wnt5a signaling pathway, and thus provide novel insights into FOXM1‐based antitumor immunotherapy.

**Abbreviations**
APC: antigen‐presenting cell
BCL‐2: B‐cell lymphoma 2
BMDCs: bone marrow‐derived dendritic cells
DC: dendritic cell
EPZ: EPZ004777
FOXM1: forkhead box transcription factor M1
IL‐12: interleukin‐12
TBM: tumor bearing mice
TME: tumor microenvironment
Tregs: T regulatory cells

## 1 Introduction

Forkhead box transcription factor M1 (FOXM1) is well known as a proliferation‐associated transcription factor with three isoforms—FOXM1a, FOXM1b, and FOXM1c. It belongs to the FOX (Forkhead box) transcription factor family, which is characterized by a conserved DNA‐binding domain referred to as the forkhead box. FOXM1 is involved in a wide array of biological functions, including cell proliferation, cell cycle regulation, angiogenesis, cell migration, tumor invasion, senescence, DNA damage repair, stem cell expansion, and renewal. Recent work has demonstrated the significance of overexpressed FOXM1 in tumor development (Bella *et al*., 2014). By positively regulating oncogenes such as VEGF and B‐cell lymphoma 2 (BCL‐2) (Karadedou *et al*., 2012; Liu *et al*., 2017b), FOXM1 impacts tumor initiation, angiogenesis, progression, drug resistance, and metastasis in prostate cancer, hepatocellular carcinoma, pancreatic cancer, colon cancer, breast cancer, and glioblastoma (Bella *et al*., 2014). Thus, FOXM1 inhibition has been suggested as an antitumor strategy (Hegde *et al*., 2011; Jiang *et al*., 2015; Ju *et al*., 2015; Wang and Gartel, 2011; Zhang *et al*., 2014).

However, deletion of FOXM1 in dendritic cells (DCs) decreased cell surface expression of major histocompatibility complex II (MHC‐II) and co‐stimulatory molecule CD86 in response to house dust mite allergen (Ren *et al*., 2013). These changes indicated that FOXM1 promotes DC maturation. DCs, the major antigen‐presenting cells (APC), play a pivotal role in antitumor immune response, including capturing and presenting tumor antigens to T cells, producing Th1 cytokines. As DCs play a pivotal role in antitumor immune response, it is questionable whether FOXM1 is a beneficial therapeutic target for cancer treatment. Investigating the effects of FOXM1 on DCs in a tumor‐specific environment is an urgently needed and a straight forward approach to determine the role of FOXM1‐mediated tumorigenesis.

The expression of FOXM1 is restricted to the cell nucleus. It has been reported that FOXM1 was tightly controlled by transcription factors and posttranslational modifications, including acetylation, sumoylation, phosphorylation, and ubiquitination (Bella *et al*., 2014; Lv *et al*., 2016; Ma *et al*., 2005; Myatt *et al*., 2014; Wang *et al*., 2010). Moreover, epigenetic modifications, including miRNA‐mediated modifications and DNA methylation, are involved in regulating FOXM1 expression (Zhang *et al*., 2012, 2017). As FOXM1 plays a critical role in carcinogenesis, exploring how FOXM1 is regulated at a cellular level may have a significant impact on the design of anticancer therapeutics.

In this work, we identified the pro‐tumorigenic function of FOXM1 by inhibiting maturation and antitumor response of bone marrow‐derived dendritic cells (BMDCs) in tumor‐bearing mice (TBM) and mimicking the tumor microenvironment (TME) through FOXM1‐Wnt family number 5A (Wnt5a) signaling. Further, we also offered evidence that FOXM1 expression was epigenetically regulated by H3K79 methylation, suggesting that FOXM1 and histone methylation are potential targets for cancer therapy.

## 2 Materials and methods

### 2.1 Animals

Male C57BL/6J and BALB/c mice were purchased from Beijing HFK Bio‐Technology Co. Ltd. Experimental colonies were maintained at the experimented animal center of Tongji Medical College (Huazhong University of Science and Technology, China) under specific pathogen‐free conditions. The mice were purchased, shipped, housed, cared for, and euthanized according to guidelines provided by the Institutional Animal Care and Use Committee of Tongji Medical College.

### 2.2 Subcutaneous xenograft model

CT‐26 and Panc02 cells were injected subcutaneously into approximately 6‐week‐old C57BL/6J and BALB/c mice to construct pancreatic cancer and colon cancer subcutaneous xenograft models. All mice were euthanized after 2 weeks of treatment.

### 2.3 Orthotopic xenograft model

Mice were anesthetized with 4% chloral hydrate and 50 μL Panc02 cell suspension at a density of 1 × 10^7^. The suspension was injected into the pancreas to construct an orthotopic xenograft model. All mice were euthanized after 2 weeks of treatment.

### 2.4 EPZ004777 and Thiostrepton treatment *in vivo*

The ectopic xenograft models were intravenously injected with EPZ004777 (EPZ) (10 mg·kg^−1^) (TargetMol T3081) or Thiostrepton (10 mg·kg^−1^) (TargetMol T1679) once every 2 days for 10 days, at which point the tumor volume was about 300 mm^3^. The tumor volume was calculated by the formula (volume = 1/2 × length × width × depth) once a day. Animals were sacrificed after 10 days of treatment, and BMDCs were generated.

### 2.5 Anti‐CD8 mAbs blocking *in vivo*

The ectopic xenograft models were injected anti‐CD8 antibody (200 μg) (clone TIB210) intraperitoneally. Then, the mice were treated by corresponding EPZ or Thiostrepton intravenously.

### 2.6 Cell culture

The pancreatic cancer cells Panc02, SW1990; colon cancer cells CT‐26, HCT116; and the normal HPDE6‐C7 cells were purchased from Shanghai Aolu biological technology Co., Ltd (Shanghai, China). All cells were maintained in Dulbecco's modified Eagle's medium (DMEM) supplemented with 10% fetal bovine serum (FBS) (Gibco, Grand Island, NY, USA, 10437028) and penicillin–streptomycin (100 IU·mL^−1^) (Gibco, 10378016) at 37 °C in a humidified 5% CO_2_ atmosphere.

### 2.7 Generation of bone marrow‐derived DCs

The BMDCs were generated from bone marrow stem cells of syngeneic mice, as previously described (Hong *et al*., 2012; Lutz *et al*., 1999). Bone marrow‐derived cells were obtained from C57BL/J6 and BALB/c mice. The cells were cultured in RPMI‐1640 medium with 10% FBS, penicillin–streptomycin (100 IU·mL^−1^) (Gibco, 10378016), GM‐CSF (20 ng·mL^−1^) (Signalway Antibody, College Park, MD, USA, AP73338), and interleukin‐4 (IL‐4, 10 ng·mL^−1^) (Signalway Antibody, AP73338) for 7 days. At day 7, semi‐adherent cells were collected as BMDCs, and the BMDC purity of >80%.

### 2.8 Treatment of cells *in vitro*

HPDE6‐C7, SW1990, Panc02, HCT116, and CT‐26 cells were cultured and treated with increasing concentrations of EPZ at dose from 0 μm to 8 μm for 72 h, then the relative IC50 values of which were calculated by spss with MTT assay based on dose–response curves. The experimental concentrations of EPZ on PDAC and colon cell lines were set according to the IC50 values of Panc02 cells and CT‐26, respectively. PDAC cell lines were cultured and treated with EPZ at increasing concentrations from 0 to 20 μm. The colon cell lines were incubated with EPZ at increasing concentrations from 0 to 10 μm. The BMDCs were treated with 1 μm EPZ, 1 μm Thiostrepton, and Wnt5a 500 ng·mL^−1^ (R&D System, Minneapolis, MN, USA). The control group comprised cells treated with DMSO. All cells were treated for 72 h before harvesting.

### 2.9 MTT assay

The pancreatic cancer and colon cancer cell viabilities were evaluated by MTT assay. The cells were seeded in 96‐well plates at a density of 1 × 10^5^ and cultured. After a 72‐h incubation with DMSO or EPZ at increasing concentrations, freshly prepared MTT (20 μL) was added to each well and incubated for 4 h at 37 °C. Thereafter, the cell medium was carefully removed, and 150 μL dimethyl sulfoxide (DMSO) was added. Cells were shaken for 10 min until no particulate matter was visible. Absorbance in each well was read at 550 nm using a multiwell spectrophotometer microplate reader. Cell viability was expressed as a percentage of that obtained with the control (untreated) cells.

### 2.10 Clonogenic assay

Cells were seeded in a 6‐well plate at 3 × 10^5^ cells confluence. Colonies were allowed to form for two weeks in medium with DMSO or increasing concentrations of EPZ. The medium was replaced every 3 days. After treatment, the cells were stained with 0.1% crystal violet for 15 min, washed three times with 1× PBS, and air‐dried. The colonies were then counted.

### 2.11 Western blotting analysis

For immunoblotting, the protein samples were separated by SDS/PAGE. Subsequently, the protein was transferred to apolyvinylidenedifluoride (PVDF) membrane (Millipore, IBFP0785C). Following, membranes were blocked with 5% no‐fat milk in TBST, and then, the membranes were incubated with specific primary antibodies at 4 °C with a 1 : 1000 dilution of anti‐FOXM1 (Santa Cruz, sc‐376471), or H3K79me2 (Cell Signaling Technology, Beverly, MA, USA, 5427S) overnight. After incubation with the relevant secondary antibodies, the reactive bands were identified using an enhanced chemiluminescence (ECL) detection reagent (Advansta, K‐12045‐D20).

### 2.12 Immunofluorescence

After incubation with or without EPZ at 1 μm, the BMDCs were stained with the anti‐FOXM1 primary antibodies followed by peroxidase‐conjugated secondary antibodies (Southern Biotech, Birmingham, AL, USA). Immunostaining was visualized using a fluorescent tyramide reagent (TSA‐direct NEL‐701, PerkinElmer, Waltham, MA, USA).

### 2.13 Quantitative RT‐PCR

Total RNA was extracted and purified using MagZol reagent (Magen, Guangzhou, China, R4801‐01) according to the manufacturer's instructions. Next, reverse transcription was performed using the transcription kit (Vazyme, Nanjing, China, R122‐01). Quantitative real‐time PCR was performed using an ABI 7900 real‐time PCR system, with all target gene primers displayed in the supporting information (Table S1). At last, data were analyzed using the 2^−ΔΔCt^ method. The mRNA levels of target genes were normalized using β‐actin detection.

### 2.14 Flow cytometry

T cells were isolated from the spleen and fluorescent conjugated antibodies including APC‐CD3 (eBioscience, San Diego, CA, USA, 17‐0032‐80), FITC‐CD4 (eBioscience, 11‐0041‐82), and PE‐CD8 (eBioscience, 12‐0081‐82). Treg cells were obtained from spleen and stained with FITC‐CD4, PE‐CD25 (eBioscience, 12‐0259‐42), and Alexa‐Foxp3 (BD, 560047). BMDCs were generated from bone marrow and incubated with the specific antibody. Antibodies used included FITC‐CD11c (eBioscience, 17‐0114‐82), APC‐CD86 (eBioscience, 11‐0862‐82), PE‐MHC‐II (eBioscience, 11‐5321‐82), Alexa‐CCR7 (eBioscience, 12‐1971‐82), and PE‐PD‐L1 (eBioscience, 12‐5982‐82). Analysis was performed on a flow cytometer (Becton Dickinson, Mountain View, CA, USA), and the FMO controls were used to set gating (Fig. S1A,B), MHC‐II‐FMO stained with CD11C and CD86, CD86‐FMO with CD11C and MHC‐II, CD4‐FMO with CD3 and CD8, CD8‐FMO with CD3 and CD4, CD25‐FMO with CD4 and Foxp3, and Foxp3‐FMO with CD4 and CD25. The cell populations were gated on forward versus side scatter pulse area and side scatter width versus height to exclude cell debris and clumps to select live singlet cells. Prepared cell samples were detected by flow cytometer immediately to make sure accurate cell counts.

### 2.15 IL‐12 p70 measurement in the supernatant of BMDCs by ELISA assay

On day 7 of culture, BMDC was treated with LPS (50 ng·mL^−1^; Sigma‐Aldrich, St. Louis, MO, USA) for 24 h (LPS priming). The supernatants were collected and detected by ELISA Kit (eBioscience).

### 2.16 Carboxyfluorescein diacetate, succinimidyl ester

T lymphocytes were isolated from the spleen of wild‐type mice. BMDCs were generated from control mice or TBM was treated with or without EPZ. The BMDCs obtained from different groups of mice were incubated with 0.5 mg·mL^−1^ mitomycin C (Sigma, 10107409001). T lymphocytes were co‐cultured at a ratio of 10 : 1 for 24 h. The proliferation of T lymphocytes was detected by Carboxyfluorescein diacetate, succinimidyl ester (CFSE) assay (ABP Bioscience, A001) with flow cytometry. The fluorescent intensity indicated the ability to promote T lymphocyte proliferation.

### 2.17 Chromatin immunoprecipitation

ChIP assays were performed with a SimpleChIP^®^ Plus Enzymatic Chromatin IP Kit (Cell Signaling Technology, 9004) according to the instruction manual. Specific antibodies against H3K79me2 and IgG were used as a control. The primers are displayed in the Supporting Information (Table S1).

### 2.18 Cell growth and TME formation

bone marrow‐derived dendritic cells from control mice were exposed to: (a) TME: 10% Panc02 or CT‐26 conditioned medium, (b) EPZ + TME, and (c) Thiostrepton +TME, respectively.

### 2.19 Bioinformatics analysis

We retrieved the dataset from the NCBI GEO databank under accession numbers http://www.ncbi.nlm.nih.gov/geo/query/acc.cgi?acc=GSE62165, http://www.ncbi.nlm.nih.gov/geo/query/acc.cgi?acc=GSE44076, http://www.ncbi.nlm.nih.gov/geo/query/acc.cgi?acc=GSE15932, and http://www.ncbi.nlm.nih.gov/geo/query/acc.cgi?acc=GSE4988 to identify the FOXM1 and DOT1L mRNA levels in pancreatic cancer and colon cancer. We next analyzed the correlation of FOXM1 and DOT1L in tumor tissues and peripheral blood using spss 20.0 software (Chicago, IL, USA). Survival data were obtained and estimated by the Kaplan–Meier method from TIMER, a web server for the comprehensive analysis of tumor‐infiltrating immune cells (Li *et al*., 2017). The histone modification tracks for humans are based on ChIP‐Seq experiments generated by the ENCODE project including 6 chip‐seq experiments: ENCSR333OPW (Target: H3K4me3), ENCSR091QXP (Target: H3K36me3), and ENCSR494CCN (Target: H3K79me2) on HCT116 cell line, while ENCSR000ASN (Target: H3K4me3), ENCSR000ASL (Target: H3K36me3), and ENCSR000ASO (Target: H3K79me2) on Homo sapiens CD14‐positive monocyte. Enriched regions were visualized on the hg19 genome with the Integrative Genomics Viewer (IGV) or UCSC genome browser (Karolchik *et al*., 2003; Thorvaldsdóttir *et al*., 2013).

### 2.20 Statistical analysis

Statistical analysis was performed using the spss 20.0 software (SPSS Software Products). All data in this study were expressed as the mean ± SD of three independent experiments. Expression of proteins determined by western blot was quantified using imagej analysis. Differences between multiple groups were examined for statistical significance using one‐way analysis of variance (ANOVA). Differences between two groups were examined for statistical significance using Student's *t*‐test. A *P*‐value less than 0.05 was considered statistically significant.

## 3 Results

### 3.1 FOXM1 was critical for BMDC maturation and function in pancreatic cancer and colon cancer

**Figure 1:**
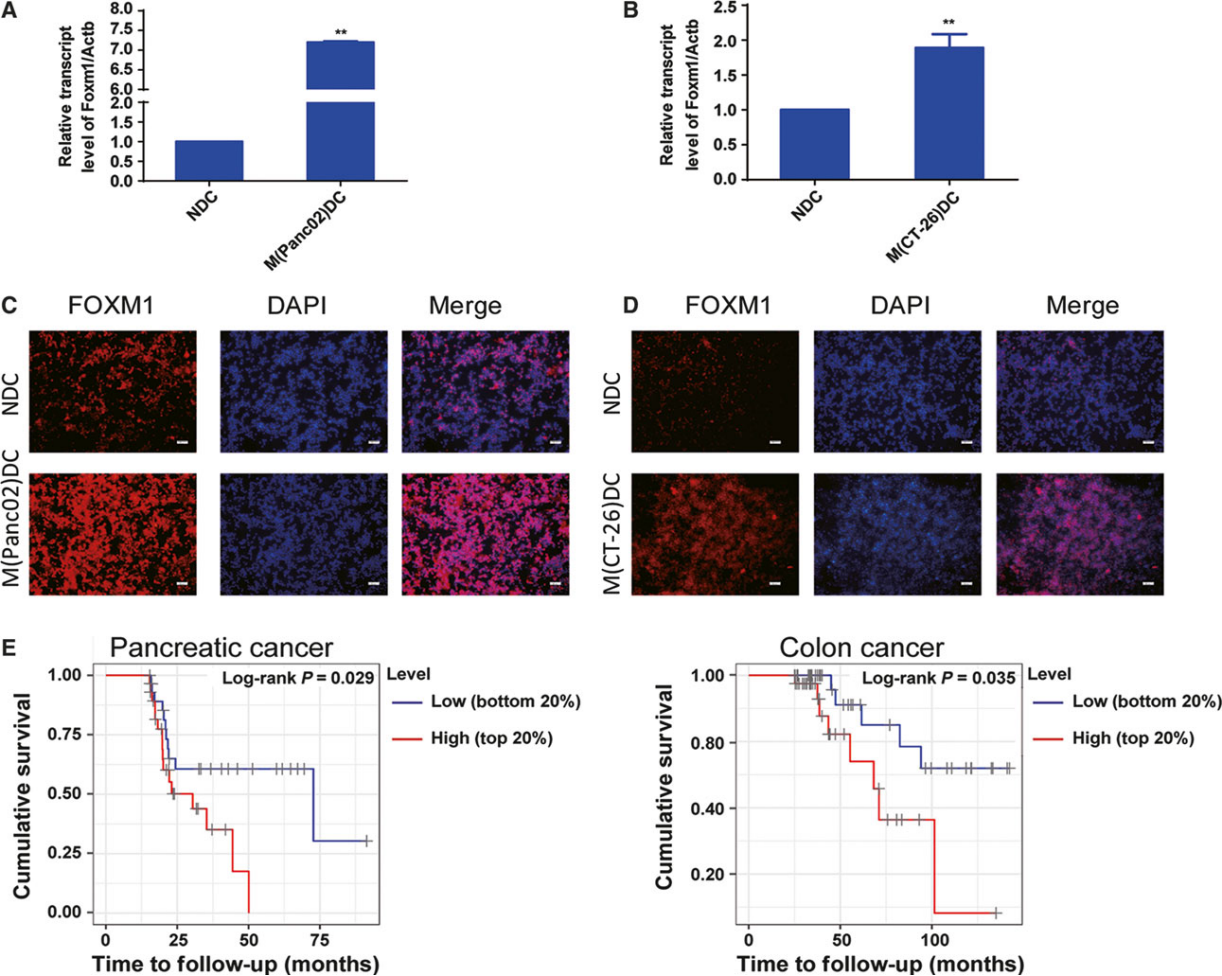
Forkhead box transcription factor M1 is overexpressed in DCs from tumor. Panc02 and CT‐26 cells were cultured and implanted (2 × 10^5^/50 μL) into the flank of C57BL/6 or BABL/c mice. Mice were euthanized 14 days after injection, and BMDCs generated by the procedure mentioned in Materials and methods. (A and B) The *Foxm1* mRNA expression of BMDCs from TBM determined by qRT‐PCR. NDC: DCs from wild‐type mice; M(Panc02)DC: DCs from Panc02 cell implanted mice; M(CT‐26)DC: DCs from CT‐26 cell implanted mice. (C and D) FOXM1 (red) level in BMDCs was determined by immunofluorescent staining. Scale bars, 50 μm. (E) Kaplan–Meier curves of pancreatic cancer and colon cancer stratified by infiltration DCs abundance from the TIMER, a web server for comprehensive analysis of tumor‐infiltrating immune cells. The ‘top 20%’ and ‘bottom 20%’ represented setting the upper or lower 20 percentiles of patient to compare. Statistical significance was calculated using multivariate Cox regression. Data represented mean ± SD from at least 3 independent experiments. **P < 0.01 compared with control.

To examine whether FOXM1 has effects on BMDCs in the tumor environment, ectopic pancreatic cancer and colon cancer mouse models were used. The C57/BL6 and BALB/c mice were inoculated with Panc02 or CT‐26 cells separately. The results showed that both mRNA expression and protein expression of FOXM1 were upregulated in BMDCs from TBM compared to that in wild‐type mice (Fig. 1A–D). Meanwhile, we detected no significant change of the expression of FOXM1 at baseline in bone marrow‐derived cells (Fig. S2A,B). Additionally, we observed overall patient survival was shorter in pancreatic cancer and colon cancer patients with a high DC infiltration abundance compared to those with low infiltration. As FOXM1 was highly expressed in TBM BMDCs, we wondered whether the shorter overall patient survival observed with a high DC infiltration was at least partly due to the high FOXM1 expression in DCs in a tumor environment (Fig. 1E).

**Figure 2:**
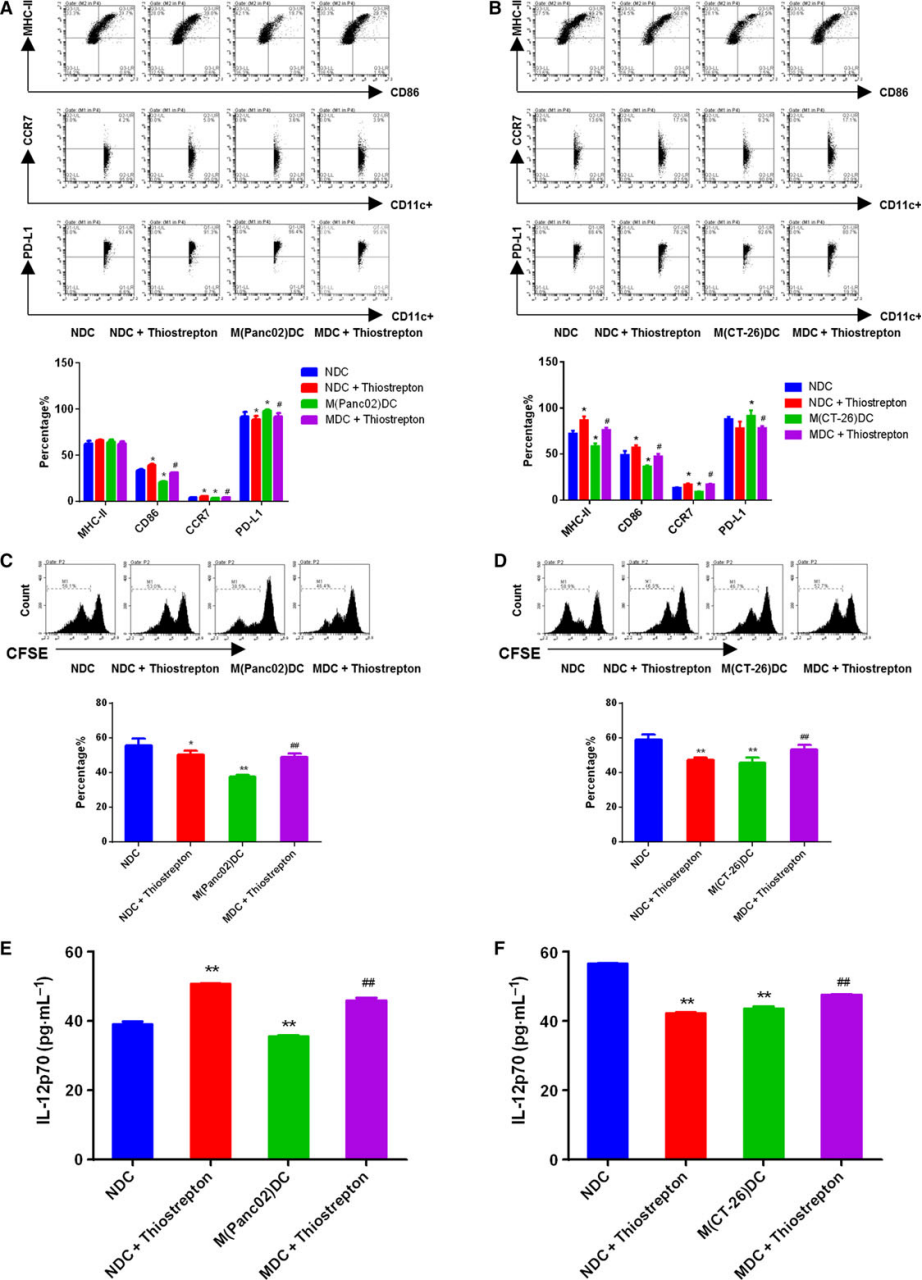
Thiostrepton promoted maturation and function of BMDCs from TBM. BMDCs were treated with Thiostrepton 1 μm for 3 days. (A and B) FACS analysis for the quantitative percentage of CD86, MHC‐II, CCR7, and PD‐L1 on gated CD11c^+^ cells in DCs from wild‐type mice, TBM. The amounts of CD11c^+^ cells set at 10 000, A is from C57BL/6J, B is from BABL/c mice. (C and D) T cells were co‐cultured with DCs at the ratio of 10 : 1 for 24 h. The proliferation on gated CD3^+^ T cells was then determined by CFSE assay. (E and F) The secretions of IL‐12 p70 from LPS primed BMDCs were quantified by ELISA assay. Data represented mean ± SD from at least 3 independent experiments.*P < 0.05, **P < 0.01, ***P < 0.001 compared with control; ^#^ *P* < 0.05, ^##^ *P* < 0.01, ^###^ *P* < 0.001 compared with the model.

Then, we cultured BMDCs from TBM with Thiostrepton, an inhibitor of FOXM1 transcriptional regulation 1 μm for 72 h *in vitro*. We found Thiostrepton treatment enhanced CD86 and CCR7 expression but decreased PD‐L1 in BMDCs, as well as normal BMDCs (Fig. 2A,B). We also co‐cultured BMDCs with splenic T cells from control mice in the ratio of 1 : 10 to determine the effects of FOXM1 on antigen presentation by BMDCs to T cells. Stimulatory capacity of BMDCs during co‐culture was assessed by measuring CFSE dilution. BMDCs from TBM treated with Thiostrepton effectively promoted T‐cell proliferation, while opposite effects were observed on normal BMDCs (Fig. 2C,D and S5C). Moreover, we evaluated the IL‐12 p70 production by BMDCs (BMDCs were treated with LPS). The inhibition of FOXM1 activity increased production of IL‐12 p70 (Fig. 2E,F). These results indicated that FOXM1 suppressed both maturation and function of BMDCs from TBM of pancreatic cancer and colon cancer.

### 3.2 DOT1L‐mediated H3K79me2 correlated with FOXM1 regulation

**Figure 3:**
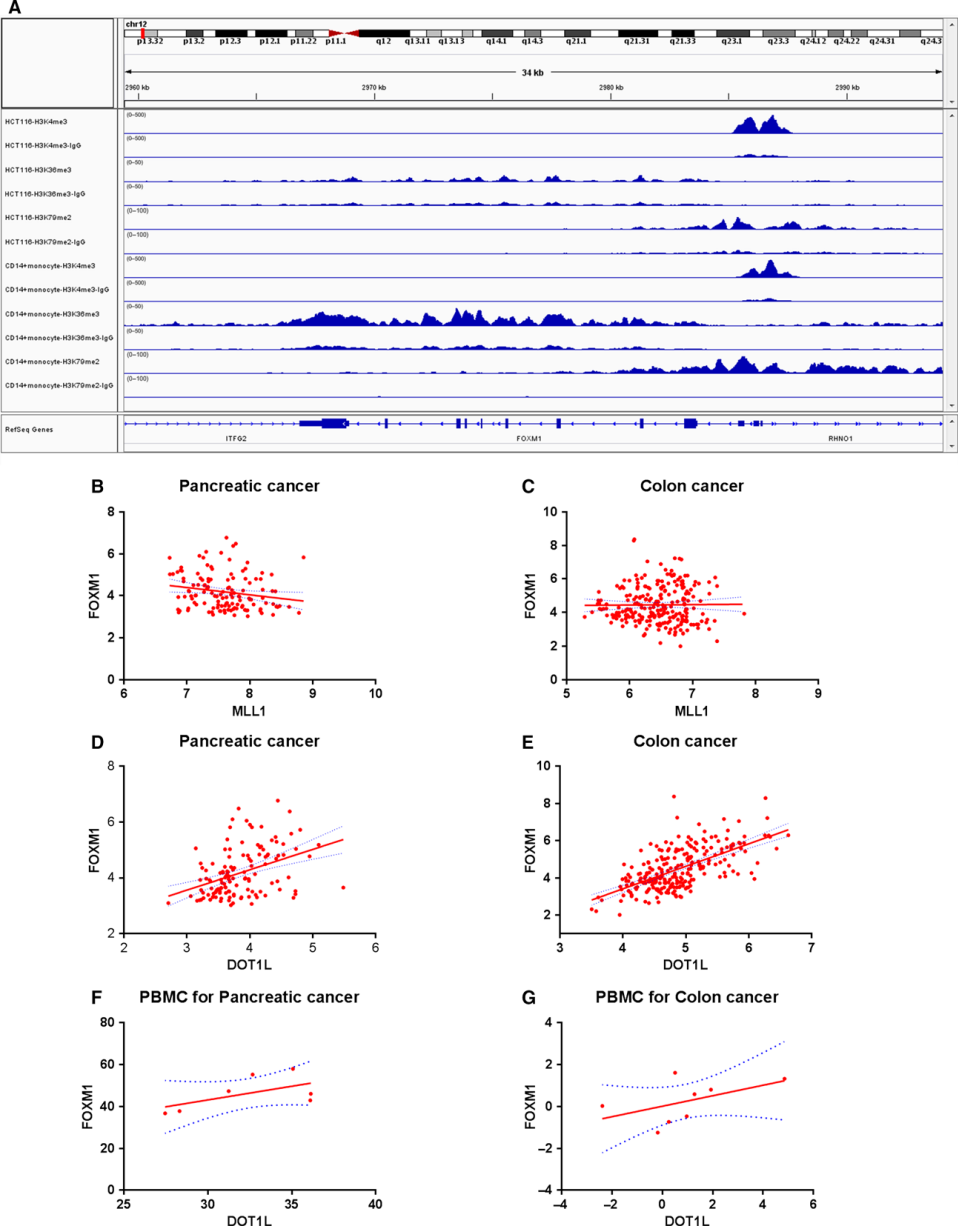
Forkhead box transcription factor M1 was correlated with DOT1L‐mediated H3K79me2 modification. (A) Representative genome‐browser view of H3K4me3, H3K36me3, H3K79me2 ChIP‐Seq for Homo sapiens colon colorectal cancer cell line HCT116 and monocytes CD14^+^ cells at the FOXM1 on chromosome 12. The Y‐axis indicated the peak height and the X‐axis indicated the genomic location. (B) and (D) The correlation between FOXM1, MLL1 (histone methyltransferase of H3K4me3), and DOT1L (histone methyltransferase of H3K79me2) transcripts in patients with pancreatic cancer was examined analyzing 131 patients (GSE62165). *R* ^2^ = 0.03541, *P* = 0.0314 (B); *R* ^2^ = 0.1615, *P* < 0.0001 (D). (C) and (E) The correlation between FOXM1, MLL1, and DOT1L transcripts in patients with colon cancer was examined analyzing 246 colon cancer patients (GSE44076). *R* ^2^ = 0.00009682, *P* = 0.8780 (C); *R* ^2^ = 0.3920, *P* < 0.0001 (E). (F) and (G) The correlation of DOT1L and FOXM1 was examined analyzing the peripheral blood of 7 pancreatic cancer patients and 8 colon cancer patients. *R* ^2^ = 0.3361, *P* = 0.1724 (F); *R* ^2^ = 0.2556, *P* = 0.2012 (G).

Next, we investigated the regulatory mechanism of FOXM1 in pancreatic cancer and colon cancer. Although gene expression is regulated by various mechanisms, histone methylation and demethylation are particularly important mechanisms (Egger *et al*., 2004). Histone methylation is associated with transcription. More specifically, tri‐methylation of H3 lysine 4 (H3K4me3), H3 lysine 36 (H3K36me3), and H3K79me2 is generally associated with transcriptional activity, while tri‐methylation of H3 lysine 9 (H3K9me3), H3 lysine 27 (H3K27me3), and H4 lysine 20 (H4K20me3) is associated with transcriptional repression (Gupta *et al*., 2010). As FOXM1 was overexpressed in pancreatic cancer and colon cancer, we focused on the H3K4me3, H3K36me3, and H3K79me2 modifications of FOXM1. Using the ‘ENCODE Transcription Factor ChIP‐Seq’ track with IGV, we analyzed the promoter (−2 kb to +2 kb relative to TSS) of FOXM1 for the presence of H3K4me3, H3K36me3, and H3K79me2 (Robinson *et al*., 2011; The, 2012; Thorvaldsdóttir *et al*., 2013). In both human colon cancer cell line HCT116‐ and CD14‐positive monocytes, we observed robust ChIP‐seq signals and peaks of H3K4me3 and H3K79me2, but not H3K36me3 on the promoter of FOXM1, suggesting that FOXM1 could be modulated through H3K4me3 and H3K79me2 modification across different cell types (Fig. 3A). Since histone methylation is modified by the histone methyltransferase, we next examined the clinical relevance of histone methyltransferase (MLL1, MLL2, MLL3, MLL4, SET1A, SET1B for H3K4me3 and DOT1L for H3K79me2) and FOXM1 in pancreatic cancer and colon cancer. We found that only the expression of disruptor of telomeric silencing 1‐like (DOT1L), the sole enzyme that specificity mediates H3K79 methylation (Min *et al*., 2003), correlated with FOXM1 in both pancreatic cancer and colon cancer tissue (Fig. 3B–E and S3A,B). These results were consistent with data obtained from peripheral blood taken from pancreatic cancer and colon cancer patients (Fig. 3F,G). Taken together, these data suggested that DOT1L‐induced H3K79me2 was associated with the regulation of FOXM1 in pancreatic cancer and colon cancer.

### 3.3 Inhibiting H3K79me2 epigenetically decreased FOXM1 in pancreatic cancer and colon cancer

**Figure 4:**
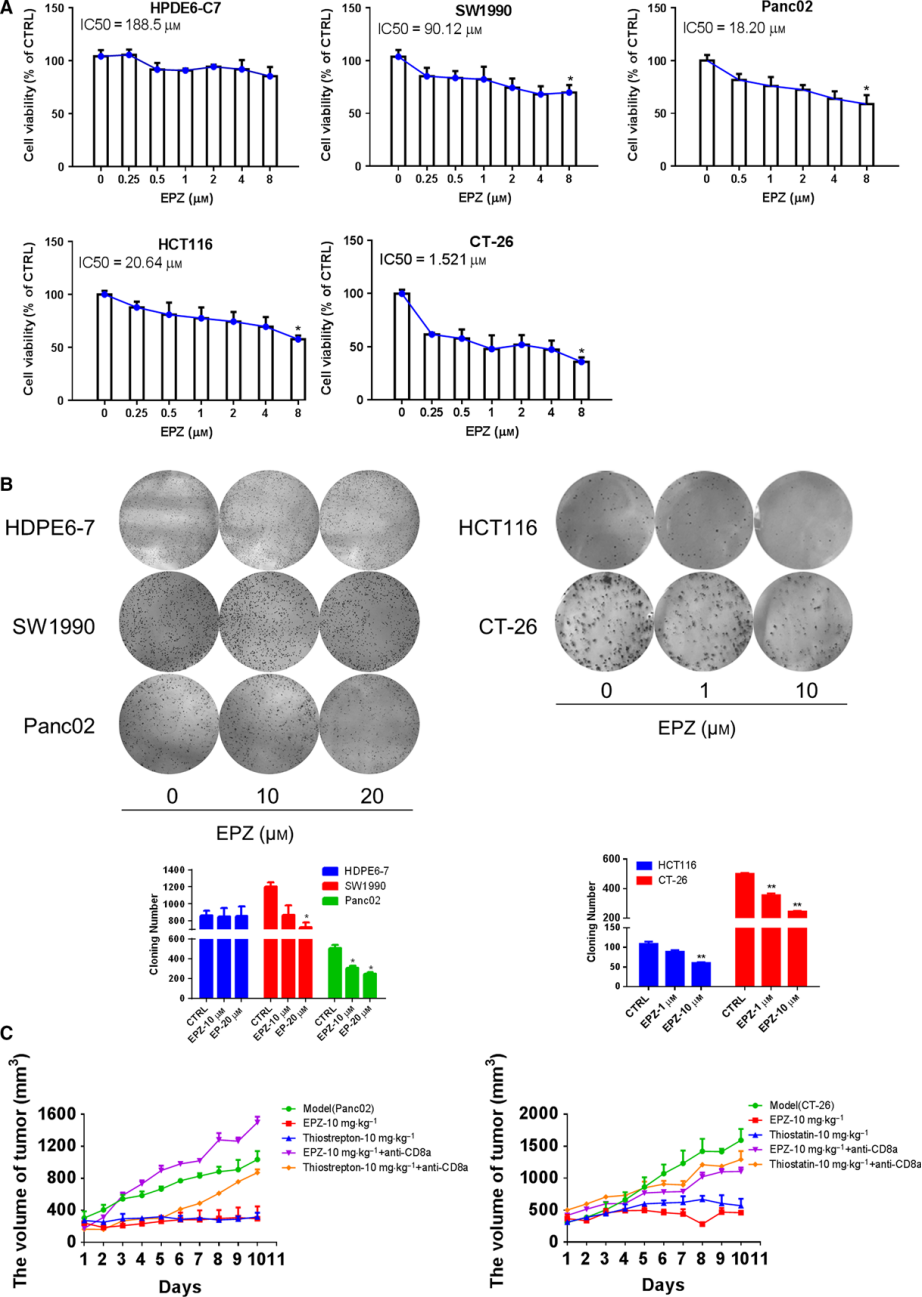
Effects of EPZ on the PDAC and colon cancer growth. (A) HPDE6‐C7, SW1990, Panc02, HCT116, and CT‐26 cells were cultured and treated with increased concentrations of EPZ from 0 μm to 8 μm for 72 h; the relative IC50 values were calculated by SPSS, shown statistical difference between 0 μm EPZ and 8 μm EPZ (*P* < 0.05). (B) Pancreatic cancer cells and colon cancer cells were treated and sustained with EPZ [PDAC cell lines (0‐20 μm), colon cell lines (0–10 μm)] for 2 weeks before stained with 0.005% crystal violet. Adjacent picture depicts the crystal violet‐stained colonies and bar graph indicated the cloning efficiency compared with untreated control. (C) Panc02 and CT‐26 cells were cultured and implanted (2 × 10^5^/50 μL) into the flank of C57BL/6J or BABL/c mice. Panc02 cells were implanted on C57BL/6 mice, while CT‐26 cells were implanted on BALB/C mice. In pancreatic cancer and colon cancer subcutaneous xenograft models, the effects of EPZ (10 mg·kg^−1^) and Thiostrepton (10 mg·kg^−1^) *in vivo* were evaluated by intravenous injection and measured the volume of tumor. Mice were sacrificed after 10 days of treatment. Data were presented as means ± SD from at least 10 mice for each experiment. *P < 0.05, **P < 0.01 compared with control.

**Figure 5:**
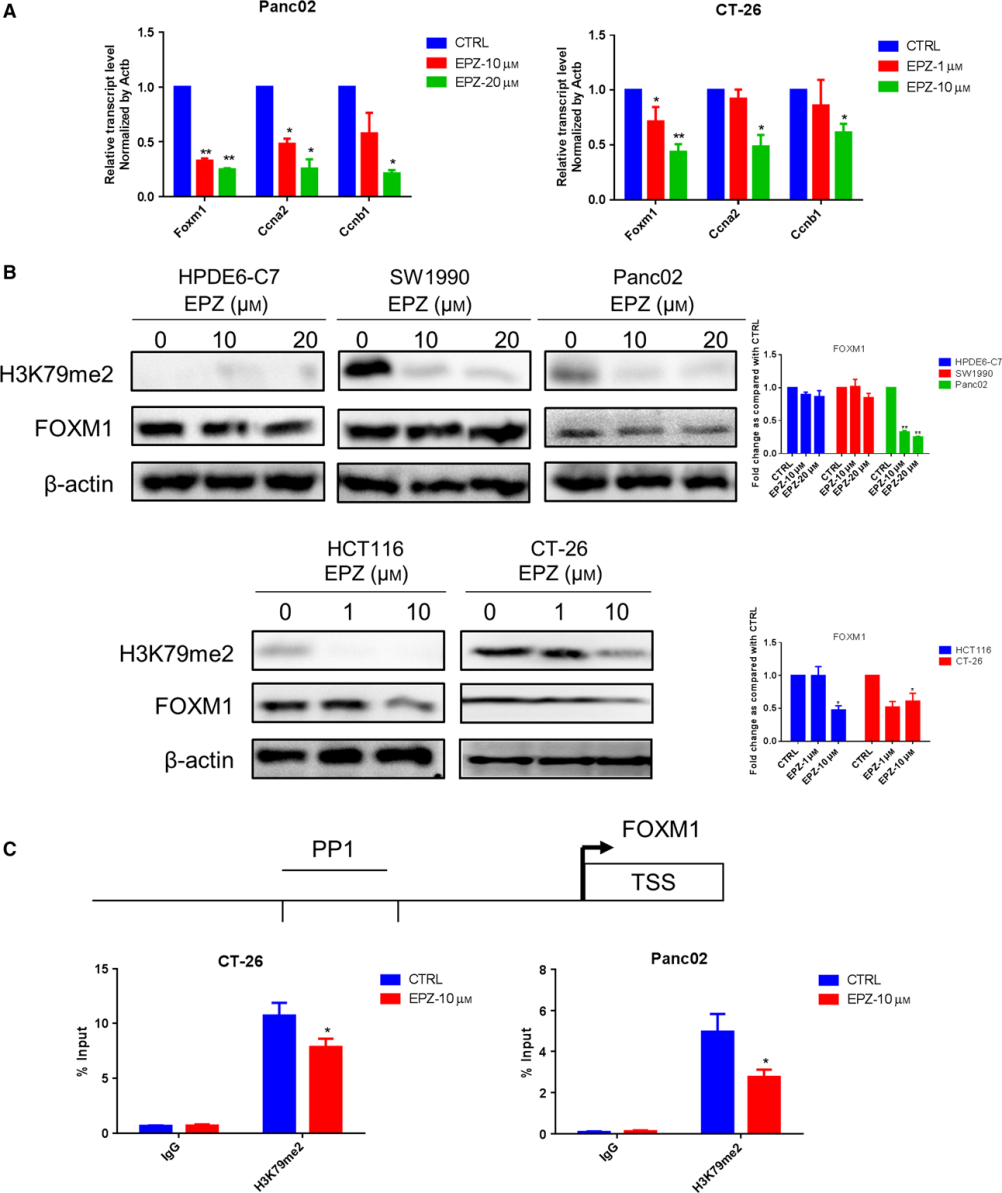
Forkhead box transcription factor M1 expression was epigenetically downregulated by EPZ in pancreatic cancer and colon cancer cells. (A) The *Foxm1* mRNA expression of Panc02 and CT‐26 cell was determined by qRT‐PCR. (B) The protein expression of FOXM1 in PDAC and colon cancer cell lines was detected by western blot. (C) A schematic representation of the mouse *Foxm1* and PP1 used for ChIP assays. ChIP assays were performed using the antibody against H3K79me2 in control and EPZ‐treated Panc02 and CT‐26 cells. Data represented mean ± SD from at least three independent experiments. *P < 0.05, **P < 0.01 compared with control.

To verify the effects of histone modification on FOXM1 expression in tumor cells, pancreatic cancer and colon cancer cells were cultured with various concentrations of EPZ for 48 h. EPZ is a small molecular inhibitor that specifically targets DOT1L. Results revealed that EPZ significantly reduced pancreatic and colon cell viability and colony‐forming activity, whereas EPZ did not significantly affect the normal pancreatic duct epithelial cells HPDE6‐C7 (Fig. 4A,B). Additionally, treatment with EPZ or Thiostrepton via intravenous tail injection reduced the tumor volumes in Panc02 or CT‐26 cells implanted xenograft murine model (Fig. 4C). Moreover, the suppression of tumor volume by EPZ and Thiostrepton was attenuated in the absence of CD8^+^ T cells, with the CD3^+^CD8^+^ T cells were 1,520 ± 18.21, 1,290 ± 10.02, significantly lower than those treated by EPZ or Thiostrepton only as 3,140 ± 34.52, 3,400 ± 15.62 (*P* < 0.001), respectively. And the results consistent with those observed in CT‐26 colon cancer model mice (*P* < 0.001) (Fig. S4A,B). Moreover, EPZ treatment remarkably reduced enrichment of H3K79me2 at the *Foxm1* promoter, while simultaneously decreasing expression of FOXM1 and its classic target genes cyclin A2 (*Ccna2*) and cyclin B1 (*Ccnb1*) (Fig. 5A–C). These data further solidified the notion that H3K79me2 modification was essential for FOXM1 expression in pancreatic cancer and colon cancer.

### 3.4 H3K79me2‐FOXM1 repressed BMDC maturation *in vivo*

**Figure 6:**
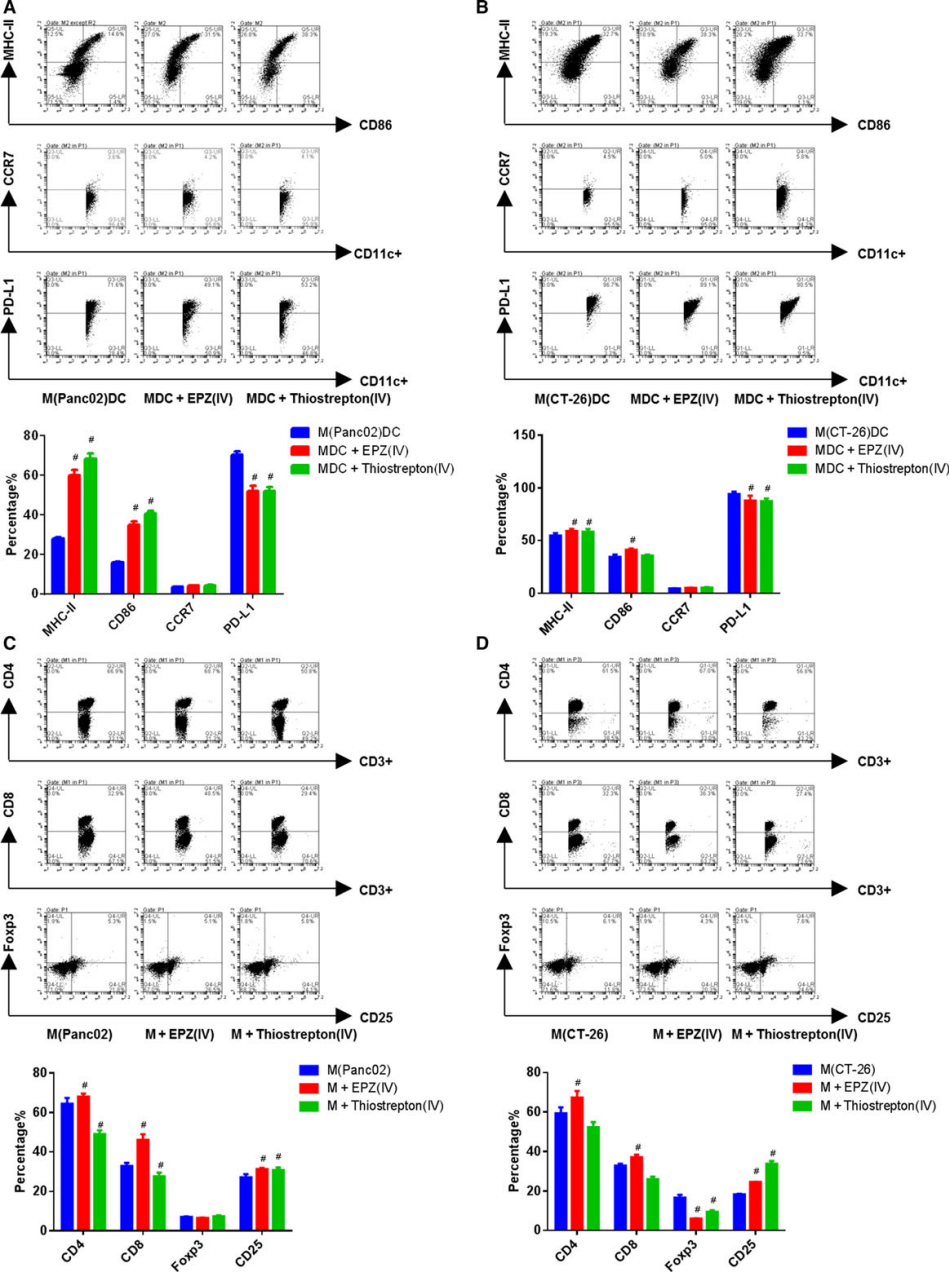
EPZ004777 and Thiostrepton improved BMDCs maturation *in vivo*. (A and B) Expressions of CD86, MHC‐II, CCR7, and PD‐L1 on gated CD11c^+^ cells in BMDCs were assessed by FACS. (C and D) The expressions of CD3, CD8, and CD4 on gated CD3^+^ T cells, and Foxp3 and CD25 on gated CD4^+^ T cells from spleen were assessed by FACS. The amounts of CD3^+^/CD4^+^ cells set at 10 000. A/C is from C57BL/6J, B/D is from BABL/c mice, each group contained at least eight mice. Data were shown as means ± SD from at least three independent experiments. ^#^ *P* < 0.05, ^##^ *P* < 0.01 compared with the model.

As FOXM1 was overexpressed in BMDCs from TBM, we hypothesized H3K79me2‐FOXM1 regulated innate immune cells in pancreatic cancer and colon cancer. To validate whether H3K79me2‐FOXM1 modulates tumor growth via alteration of the TME composition *in vivo*, we measured T cells from spleen including CD4^+^, CD8^+^, and regulatory T cell (Tregs) as well as BMDCs from EPZ‐ or Thiostrepton‐treated TBM by tail vein injections. Interestingly, we found both EPZ and Thiostrepton treatment enhanced MHC‐II and co‐stimulatory molecule CD86 expression on BMDCs from TBM (Fig. 6A,B), which was consistent with our finding on normal BMDCs (Fig. S5A,B). In addition, the expression of CCR7 increased, while PD‐L1 decreased (Fig. 6A,B). We also found that EPZ raised the population of CD4^+^ and CD8^+^ T cells to 68.7% (6,870 ± 77.21) and 48.5% (4,850 ± 82.61) compared to those in the Panc02 pancreatic cancer model as 66.9% (6,690 ± 78.52, *P* < 0.05) and 32.9% (3,290 ± 27.72, *P* < 0.05), but slightly lowered the differentiation of Tregs to 5.1% (510 ± 12.81) versus those in the Panc02 pancreatic cancer model as 5.3% (530 ± 17.01). The consistent results were shown in CT‐26 colon cancer mice. However, Thiostrepton had the opposite effects on T cells (Fig. 6C,D). These data suggest that H3K79me2 modification represses BMDC maturation through FOXM1 and affected T cells in a FOXM1‐independent manner *in vivo*.

### 3.5 H3K79me2‐FOXM1 inhibited maturation and attenuated function of BMDC *in vitro*

**Figure 7:**
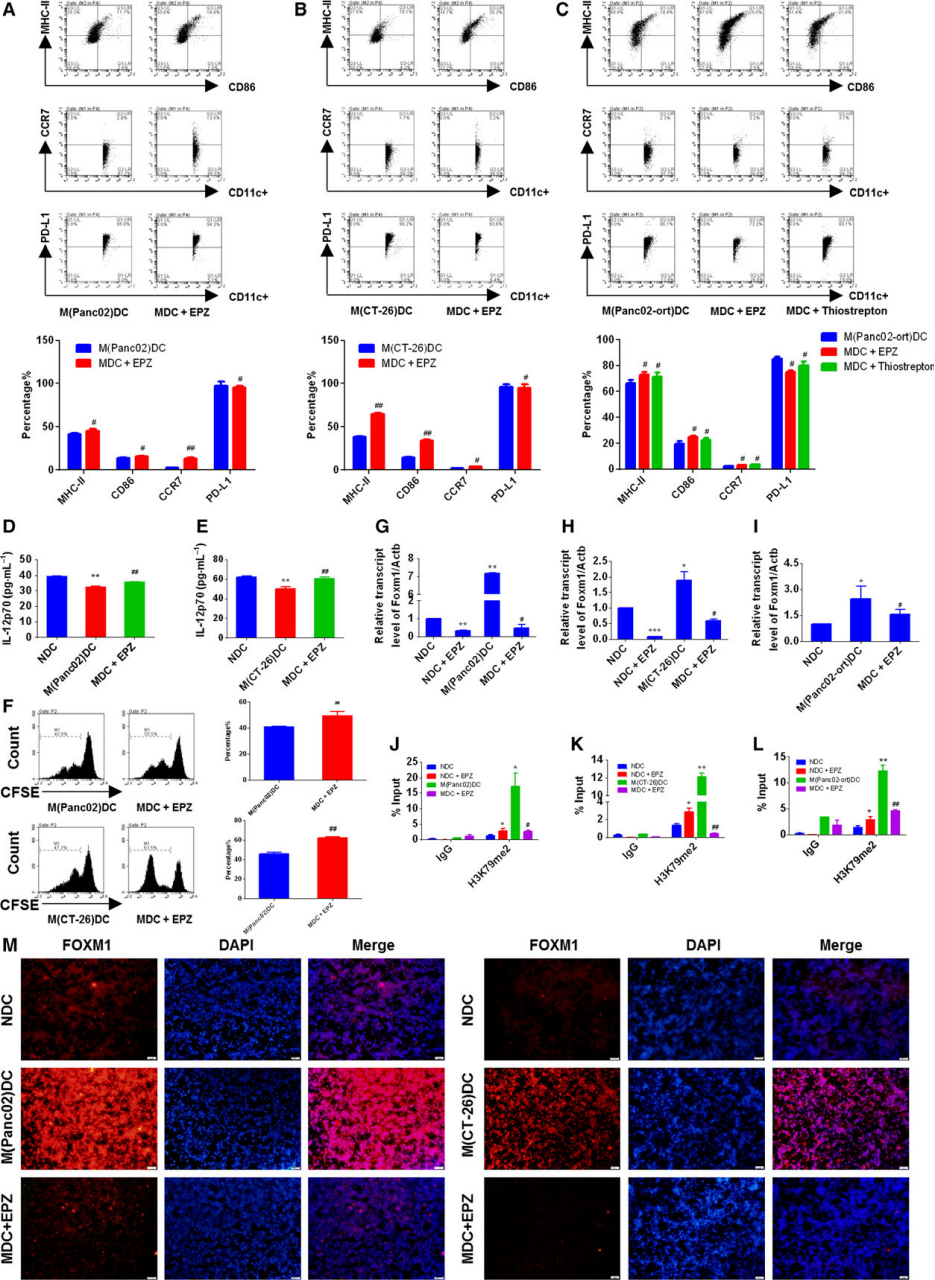
The EPZ promoted BMDCs maturation and function by inhibiting FOXM1. (A and C) FACS analysis for the quantitative percentage of CD86, MHC‐II, CCR7, and PD‐L1 on gated CD11c^+^ cells in BMDCs from orthotopic or ectopic mice model. (D and E) The secretions of IL‐12 p70 from LPS primed BMDCs were quantified by ELISA assay. (F) T cells were co‐cultured with DCs at the ratio of 10 : 1 for 24 h, and then, the proliferation on gated CD3^+^ T cells was determined by CFSE assay. (G–I) The *Foxm1* mRNA expression of BMDCs was determined by qRT‐PCR. (J–L) ChIP assays were performed using the antibody against H3K79me2 at *Foxm1* promoter in BMDCs. (M) The protein expression of FOXM1 in BMDCs was detected by immunofluorescent staining. Scale bars, 50 μm. Data represented mean ± SD from at least three independent experiments.*P < 0.05, **P < 0.01, ***P < 0.001 compared with control; ^#^ *P* < 0.05, ^##^ *P* < 0.01 compared with the model.

To further investigate the effect of H3K79me2‐FOXM1 on BMDCs, we collected BMDCs from TBM, including orthotopic and ectopic xenograft models, and incubated them with EPZ (1 μm) and Thiostrepton (1 μm) for 72 h *in vitro*. With FACS analysis, we found the treatment increased expression of MHC‐II, CD86, and CCR7, but lowered PD‐L1 in BMDCs (Fig. 7A–C). Moreover, we evaluated the IL‐12 p70 production of BMDCs (BMDCs were treated with LPS) and found inhibition of H3K79me2 also increased production of IL‐12 p70 (Fig. 7D,E). Additionally, BMDCs effectively promoted T‐cell proliferation when treated with EPZ (Fig. 7F), consistent with our findings on normal BMDCs (Fig. S5C).

We next performed ChIP assays to investigate whether H3K79me2 epigenetically affected Foxm1 expression in BMDCs. As expected, enrichment of H3K79me2 at the Foxm1 promoter in BMDCs from the orthotropic and ectopic models was much higher than those of BMDCs from wild‐type mice. Additionally, H3K79me2 enrichment was significantly decreased by EPZ treatment (Fig. 7J–L). Consistent with its effects on the enrichment of H3K79me2 modification, EPZ treatment reduced *Foxm1* mRNA and protein expression in BMDCs (Fig. 7G–I and M). Together, these data indicated that H3K79 methylation epigenetically upregulated FOXM1 to inhibit maturation and function of BMDCs.

### 3.6 Tumor‐conditioned medium inhibited BMDC maturation via H3K79me2‐FOXM1

**Figure 8:**
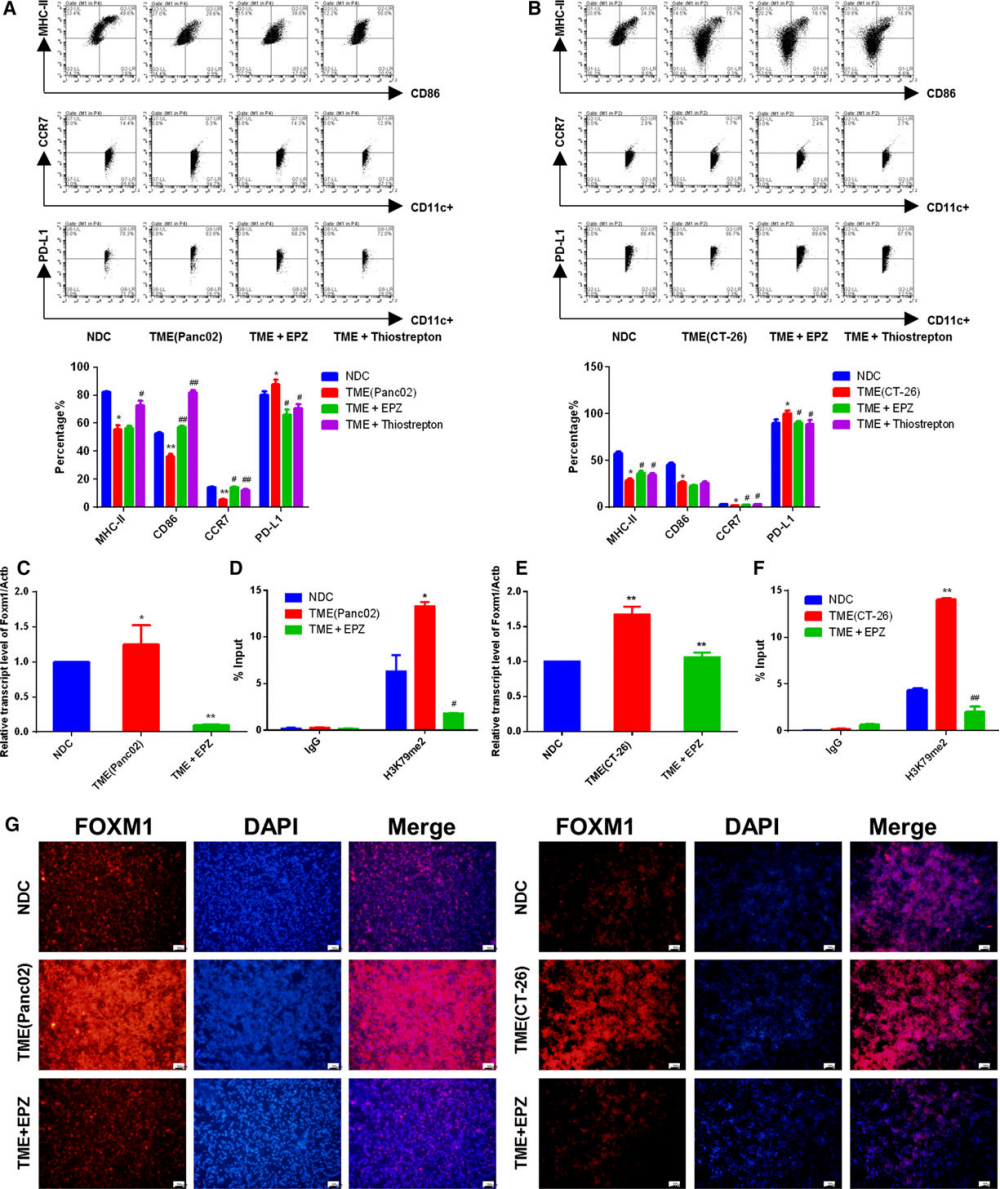
The supernatant of cancer cells inhibited BMDCs maturation via H3K79me2‐FOXM1. (A and B) The expression levels of CD86, MHC‐II, CCR7, and PD‐L1 on gated CD11c^+^ cells in BMDCs were assessed by FACS. NDC: BMDCs from wild‐type mice; TME(Panc02): Culture medium from Panc02 cells was added to NDC; TME + EPZ: Culture medium from cancer cell and EPZ (1 μm) was added to NDC; TME + Thiostrepton: Culture medium from cancer cell and Thiostrepton (1 μm) was added to NDC; TME(CT‐26): Culture medium from CT‐26 cells was added to NDC. (C) and (E) The *Foxm1* mRNA expression of BMDCs was determined by qRT‐PCR. (D) and (F) ChIP assays were performed using the antibody against H3K79me2 at *Foxm1* promoter in BMDCs. (G) The protein level of FOXM1 was determined by immunofluorescent staining. Scale bars, 50 μm. Data represented mean ± SD from at least three independent experiments.*P < 0.05, **P < 0.01, compared with control; ^#^ *P* < 0.05, ^##^ *P* < 0.01 compared with the model.

Dendritic cells play an important role in both tumorigenesis and tumor repression by exerting differential pro‐tumorigenic and antitumorigenic functions depending on the local microenvironment. Based on our previous work, and that of other labs, DC dysfunction in tumors might be a consequence of soluble factors secreted by cancer cell into the TME. These soluble factors include Reg3 g, IL‐6, and IL‐10 in tumor‐conditioned medium (Liu *et al*., 2017a; Wang *et al*., 2016). To elucidate whether tumor‐conditioned medium suppressed BMDC maturation via elevated H3K79me2‐FOXM1 expression, we conducted an *in vivo* experiment pretreating BMDCs from wild‐type mice with conditioned medium from Panc02 or CT‐26 cells, mimicking TME, before pulsing them with EPZ or Thiostrepton. We found that BMDCs cultured with tumor‐conditioned serum had lower MHC‐II, CD86, and CCR7 expression accompanied by higher levels of PD‐L1 compared with the control group. Notably, inhibition of BMDC maturation and function was partly reversed by treatment with EPZ and Thiostrepton (Fig. 8A,B).

Moreover, ChIP assays demonstrated that EPZ treatment significantly abrogated the enriched H3K79 methylation at the FOXM1 promoter induced by cancer serum in BMDCs. Abrogated H3K79 methylation was accompanied by decreased FOXM1 expression at both the mRNA and protein levels (Fig. 8C–G). These data further speculated that tumor‐conditioned medium impaired BMDC maturation was associated with H3K79me2‐FOXM1 upregulation. Therefore, targeted removal of inhibitory soluble factors from tumor‐conditioned medium may unleash the full potential of BMDCs to trigger antitumor immunity in pancreatic cancer and colon cancer.

### 3.7 FOXM1 inhibited BMDC maturation through Wnt5a pathway

**Figure 9:**
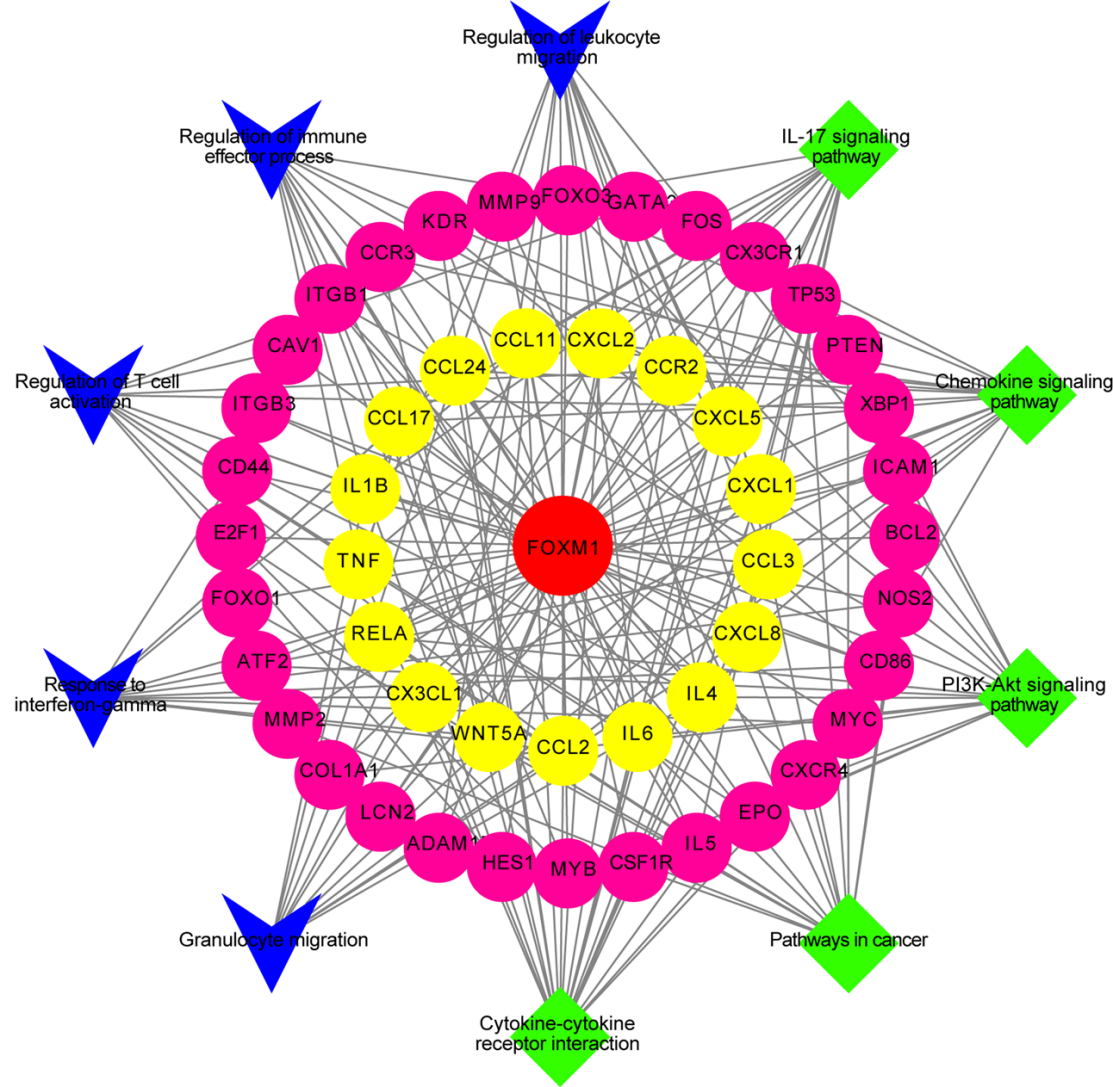
Candidate target gene pathway/immune function network of FOXM1. There were 48 candidate genes, five core pathways, and five immune functions which were validated in published literatures. Diamond represented pathways; Vee represented immune functions; circle represented target genes; center circle represented FOXM1. Target gene in the inner circle showed much more interactions with candidate ingredients than those in the outer circles.

**Figure 10:**
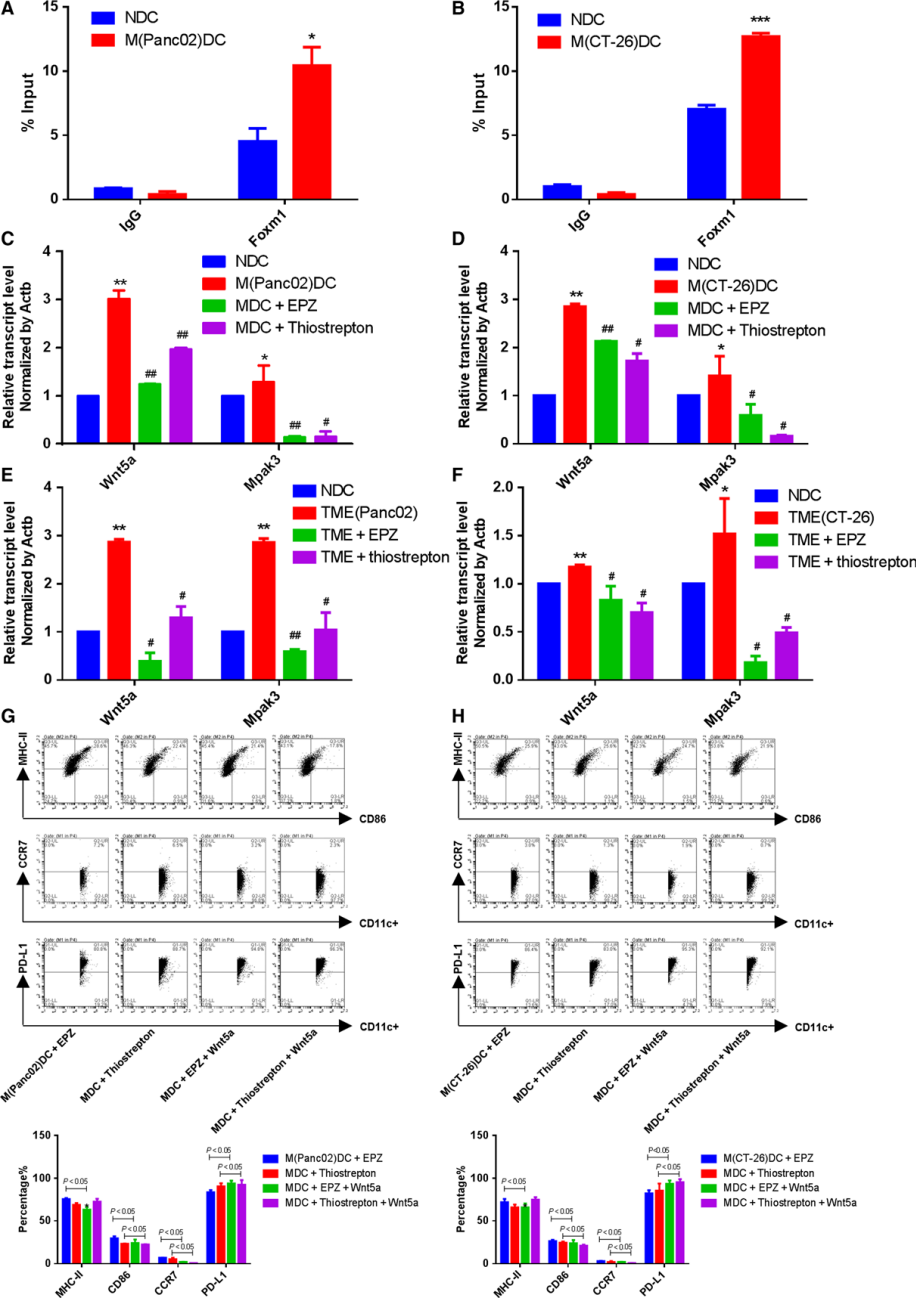
Forkhead box transcription factor M1 inhibited BMDCs maturation through Wnt5a pathway. (A and B) ChIP assays were performed using the antibody against FOXM1 at *Wnt5a* promoter in BMDCs. (C and D) The *Wnt5a* expression and *Mapk3* mRNA expression of BMDCs were determined by qRT‐PCR. (E and F) The *Wnt5a* expression and *Mapk3* mRNA expression of BMDCs cultured with medium from cancer cells were determined by qRT‐PCR. (G and H) The expression levels of CD86, MHC‐II, CCR7, and PD‐L1 on gated CD11c^+^ cells in BMDCs were assessed by FACS. MDC+EPZ+Wnt5a: BMDCs from TBM were treated with exogenous Wnt5a in the presence of EPZ. MDC + Thiostrepton + Wnt5a: BMDCs from TBM were treated with exogenous Wnt5a in the presence of Thiostrepton. Data represented mean ± SD from at least three independent experiments.*P < 0.05, **P < 0.01, compared with control; ^#^ *P* < 0.05, ^##^ *P* < 0.01 compared with the model.

Above data suggested that H3K79me2‐FOXM1 is essential for BMDC maturation and function during the antitumor response to pancreatic cancer and colon cancer. We next explored the major target of FOXM1 in BMDCs. Using cytoscape (Shannon *et al*., 2003), we constructed a candidate FOXM1‐target gene pathway/immune function network depicting the relationship between FOXM1 target genes, related pathways, and immune function (Fig. 9). In this study, we selected the core components of the network with degree values greater than four for further research. Then, we focused on WNT5A, the known target gene of FOXM1, which was reported as an inhibitor of DC maturation (Bergenfelz *et al*., 2013). Increased FOXM1 enrichment was detected with a ChIP assay at the Wnt5a promoter in BMDCs from TBM (Fig. 10A,B). Moreover, the expression of Wnt5a was upregulated in BMDCs from TBM compared to wild‐type mice and strongly attenuated by treatment with both inhibitors. Upregulation of the downstream gene *Mapk3* was also attenuated by EPZ and Thiostrepton (Fig. 10C,D). Consistent results were detected in BMDCs from wild‐type mice incubated with Panc02 or CT‐26 cell‐conditioned medium and treated with EPZ and Thiostrepton (Fig. 10E,F)**.** Additionally, exogenous Wnt5a expression reduced BMDCs maturation in the presence of EPZ or Thiostrepton (Fig. 10G,H). These data indicated that H3K79me2‐FOXM1 represses BMDC maturation through the Wnt5a pathway.

## 4 Discussion

By applying subcutaneous and orthotopic cancer murine models and an *in vitro* cell culture system mimicking the TME, we have demonstrated that H3K79me2‐FOXM1 plays a crucial role in accelerating pancreatic cancer and colon cancer progression by attenuating antitumor responses including BMDC maturation, cytokine secretion, and T‐cell activation.

Forkhead box transcription factor M1 plays an important role in biological progresses, including cell proliferation, cell migration, cell invasion, and DNA damage repair (Wang *et al*., 2010). A growing body of literature strongly suggests that abnormal upregulation of FOXM1 is a hallmark of human malignancies (Wang *et al*., 2010; Wierstra and Alves, 2007). In this study, we showed that FOXM1 is a suppressor of BMDC maturation in pancreatic cancer and colon cancer. Increased expression of FOXM1 was observed in BMDCs from TBM. Moreover, inhibiting activity of FOXM1 upregulated CD86 and CCR7, but lowered PD‐L1 on the BMDC surface. The inhibition of FOXM1 also increased IL‐12 p70 production and promoted T‐cell proliferation. Additionally, high infiltration in DCs correlated with poor survival in pancreatic cancer and colon cancer patients. Therefore, our work indicated that FOXM1 inhibited both maturation of BMDCs and their tumor‐suppressing function while promoting tumorigenesis. However, previous work showed that FOXM1 promotes allergen‐induced lung inflammation by inducing goblet cell metaplasia, increasing recruitment of eosinophils and macrophages to the lung, and increasing the cell surface expression of MHC‐II and CD86 (Ren *et al*., 2013). The established role of FOXM1 in DCs appears contradictory to our findings at first, but the different immune microenvironments make both possible. In allergen‐induced lung inflammation, the immune activation was excessive, while the immune response was seriously weakened in a tumor immune microenvironment. Therefore, it appears that the role of FOXM1 as a DC maturation suppressor or promoter is highly immune environment‐specific.

Forkhead box transcription factor M1 is tightly controlled at both the transcriptional and posttranslational levels. Its promoter contains many transcription factors. E2F1, ERα, HIF‐1α, c‐Myc, and STAT3 are reported as transcriptional activators of FOXM1 (Blanco‐Bose *et al*., 2008; Horimoto *et al*., 2011; Kryczek *et al*., 2014; de Olano *et al*., 2012; Xia *et al*., 2009), while C/EBPα and ERb1 act as transcriptional repressors of the FOXM1 promoter (Horimoto *et al*., 2011; Wang *et al*., 2007). It is well known that epigenetic modifications are involved in FOXM1 transcriptional regulation, including miRNA‐mediated regulation and DNA methylation (Zhang *et al*., 2012, 2017). In addition, FOXM1 function is regulated by posttranslational modifications, including acetylation, sumoylation, phosphorylation, and ubiquitination. With bioinformatics analysis, we suggested that DOT1L‐induced H3K79me2 is associated with FOXM1 regulation in pancreatic cancer and colon cancer. Moreover, inhibition of FOXM1 or H3K79me2 could suppress tumor development in TBM, while the absence of CD8^+^ T cells in TBM abolished the effect. This suggested that the antitumor effects were partly due to the CD8^+^ T cells, which were educated by DCs. Our findings reveal that FOXM1 is epigenetically regulated by H3K79 methylation modification in both tumor cells and BMDCs. H3K79me2 enrichment was detected on the FOXM1 promoter. DOT1L suppressed this H3K79me2 enrichment, but also decreased expression of FOXM1. The DOT1L inhibitor also partially reversed immune suppressive effects of FOXM1 on BMDCs. Our findings on FOXM1 transcriptional regulation by histone methylation contribute to the known epigenetic regulating mechanisms for FOXM1. Furthermore, considering that H3K79me2 enrichment of the FOXM1 promoter was detected in additional cell and tissue types, outside of the BMDCs, and pancreatic cancer and colon cancer cells addressed in this study (Figs 3A and S6), we suggested that similar mechanisms might broadly be applicable to other tissues. It would be necessary to be concerned with multitargets or side effect on the clinical therapy by small molecular inhibitors to histone methylation.

During initiation and tumorigenesis, the immune response was gradually attenuated due to weak tumor immunogenicity and immunosuppressive factors. These factors were produced by both tumor and tumor‐associated cells including Tregs and myeloid‐derived suppressor cells (MDSC) (Wang *et al*., 2016). DCs, critical regulators of host immunity, serve as a bridge between the innate and adaptive immune responses. DCs also play a relevant role in tumorigenesis by exerting differential pro‐tumorigenic and antitumorigenic functions, depending on the local microenvironment (Legitimo *et al*., 2014). In TME, the APC function of DCs is suppressed by soluble factors secreted from tumor cells and tumor‐associated cells. It has been reported that IL‐10 irreversibly blocked monocyte‐to‐DC differentiation. Moreover, vascular endothelial growth factor (VEGF), Reg3 g, gangliosides, prostanoids, and polyamines secreted by tumor cells could inhibit differentiation and maturation of DCs (Fricke and Gabrilovich, 2006; Pinzon‐Charry *et al*., 2005). Previous research in breast cancer indicated that IL‐10 and IL‐6 inhibit DC maturation through repression of miR‐155 upregulation (Wang *et al*., 2016). Our work revealed that tumor‐conditioned medium inhibits BMDC maturation via upregulation of H3K79me2 on the FOXM1 promoter, which increases FOXM1 expression in pancreatic cancer and colon cancer. It remains to be determined whether H3K79me2 and miR‐155 participate in the regulation of DC maturation during antitumor immunity. The underlying molecular mechanisms, however, need further elucidation.

As a key proliferation‐associated transcription factor, FOXM1 regulates the expression of over 220 genes that are involved in various biological processes (Lv *et al*., 2016). By constructing a FOXM1‐target gene pathway/immune function network, we selected Wnt5a as a candidate target gene for FOXM1 in BMDC maturation and function. Our study further revealed that FOXM1 directly upregulated Wnt5a, a downstream gene of FOXM1, in BMDCs. Furthermore, exogenous Wnt5a abrogated the effects on BMDC maturation of FOXM1 or H3K79me2 inhibition. As Wnt5a is both an effector of FOXM1 and an inhibitor of DC maturation (Bergenfelz *et al*., 2013), it is fitting that our results indicate that FOXM1 inhibits maturation of BMDCs, at least in part, through the Wnt5a pathway.

Furthermore, our study was the first to demonstrate that H3K79me2 significantly contributed to BMDC maturation and function. DOT1L inhibition has been suggested as an antitumor strategy for the development of active immunotherapies using tumor antigen‐loaded DCs, but exploration of this avenue has become stagnant (Stein and Tallman, 2015; Vlaming and van Leeuwen, 2016; Xu and Cao, 2011). Our study indicates that H3K79me2 targeting may provide a meaningful alternative for DC‐based immunotherapies.

## 5 Conclusions

**Figure 11:**
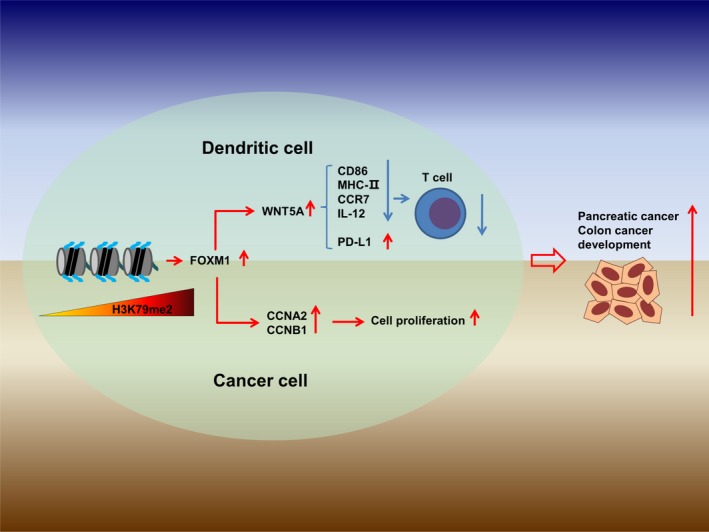
H3K79me2 FOXM1 accelerated tumor growth. The FOXM1 expression was epigenetically upregulated by H3K79me2 modification, promoted pancreatic and colon tumor progression, and inhibited antitumor immunity through Wnt5a.

In summary, our findings revealed an important epigenetic modification to FOXM1, which affects its novel tumor‐promoting role in tumor immunity. We demonstrated that the H3K79me2 modification upregulated transcription of FOXM1. Increased FOXM1 enhanced tumorigenesis by upregulating CCNA2 and CCNB1 in tumors. FOXM1 also suppressed the maturation of BMDCs via direct activation of Wnt5a and weakened promotion of T‐cell proliferation. Overall, increased FOXM1 resulted in accelerated tumor growth (Fig. 11).

## Author contributions

MX participated in the study design, material support, coordination, and supervision of the study. ZZ designed the experimental validation, performed experiments, analyzed the data, and drafted the manuscript. HC and RX performed experiments and analyzed the data. SL, QX, NX, QC, YQ, RH, and ZS performed the experiments. HW participated in the study design and guidance of the study. All authors read and approved the final manuscript.

## Conflict of interest

The authors declare no conflict of interest.

## Supporting information


**Fig. S1.** Gating strategy used to define BMDCs and T cells populations.

**Fig. S2.** The expression of FOXM1 at basic line.

**Fig. S3.** The correlation between the histone methyltransferase and FOXM1 in pancreatic cancer and colon cancer.

**Fig. S4.** Anti‐CD8a inhibited CD8 +  T cell population *in vivo*.

**Fig. S5.** EPZ and Thiostrepton improved normal BMDCs maturation.

**Fig. S6.** H3K79me2 modification tracks in human.

**Table S1.** The sequences of qRT‐PCR primers.
