# Supplementary figures and images for: Epigenetically modulated FOXM1 suppresses dendritic cell maturation in pancreatic cancer and colon cancer
Source: Mol Oncol. 2019 Feb 15;13(4):873–93. doi: 10.1002/1878-0261.12443 (PMC6441919; doi:10.1002/1878-0261.12443)

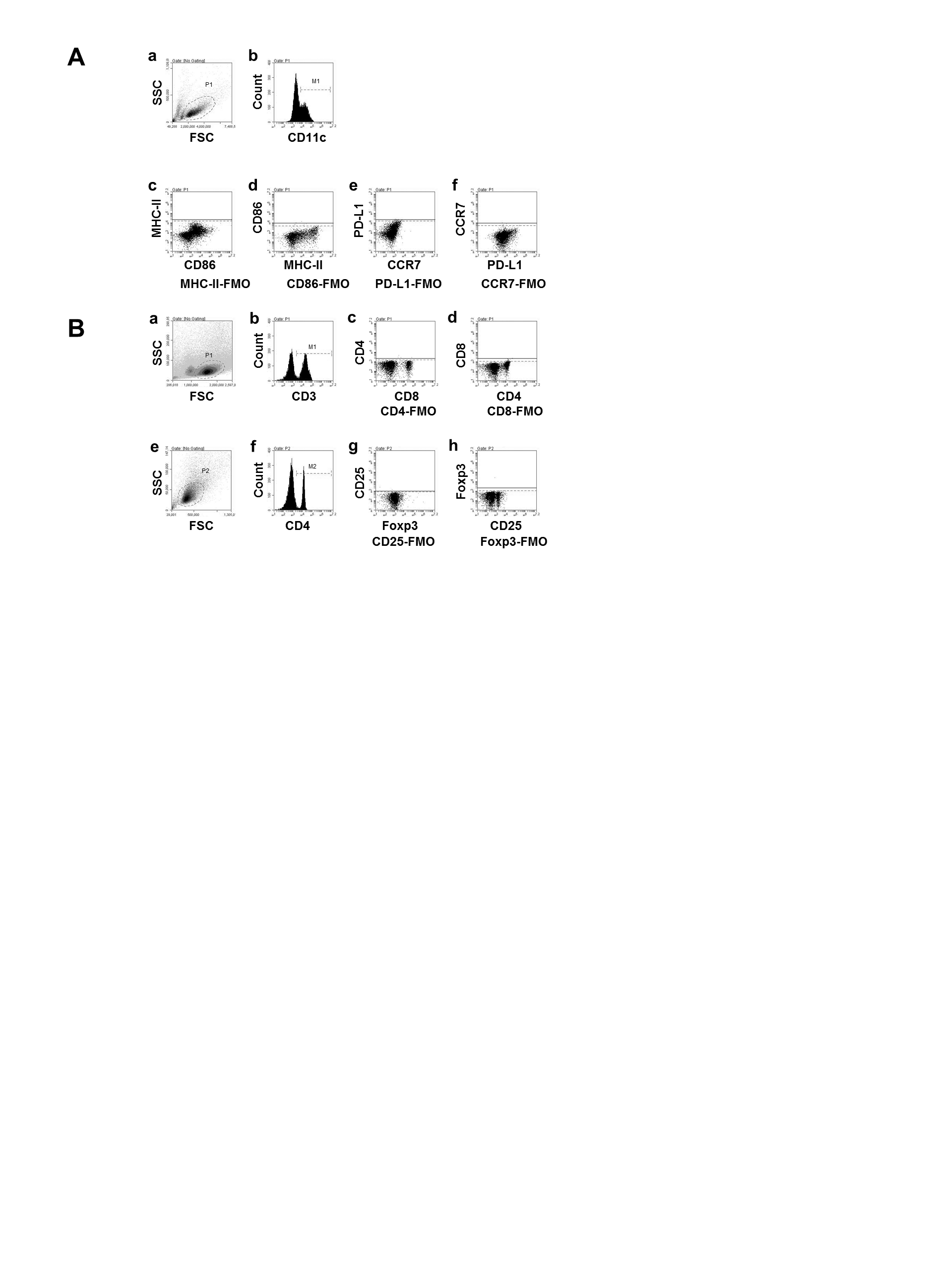

Supplement: Supplementary file 1 — Fig. S1. Gating strategy used to define BMDCs and T cells populations. [file MOL2-13-873-s001.tif]

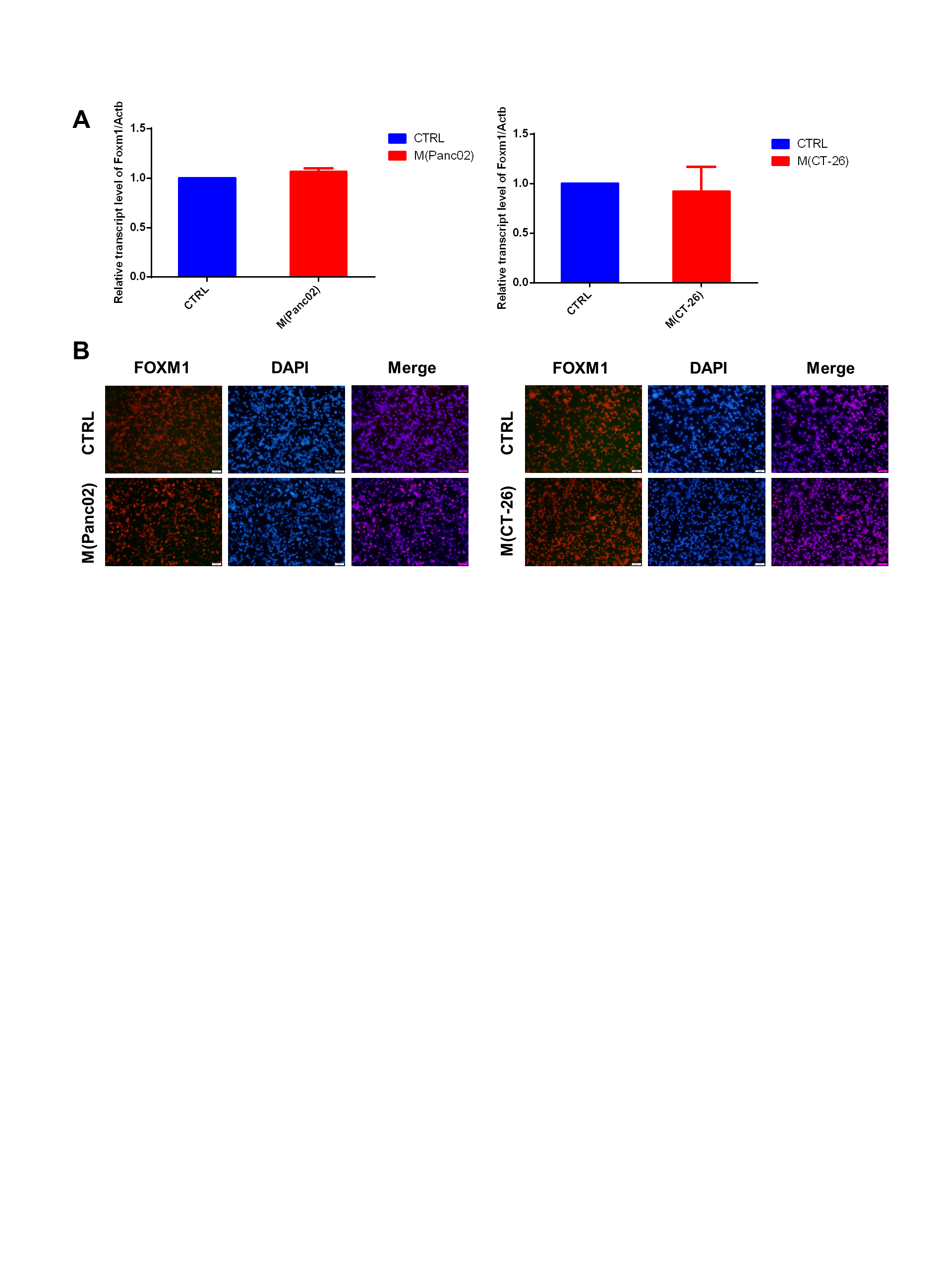

Supplement: Supplementary file 2 — Fig. S2. The expression of FOXM1 at basic line. [file MOL2-13-873-s002.tif]

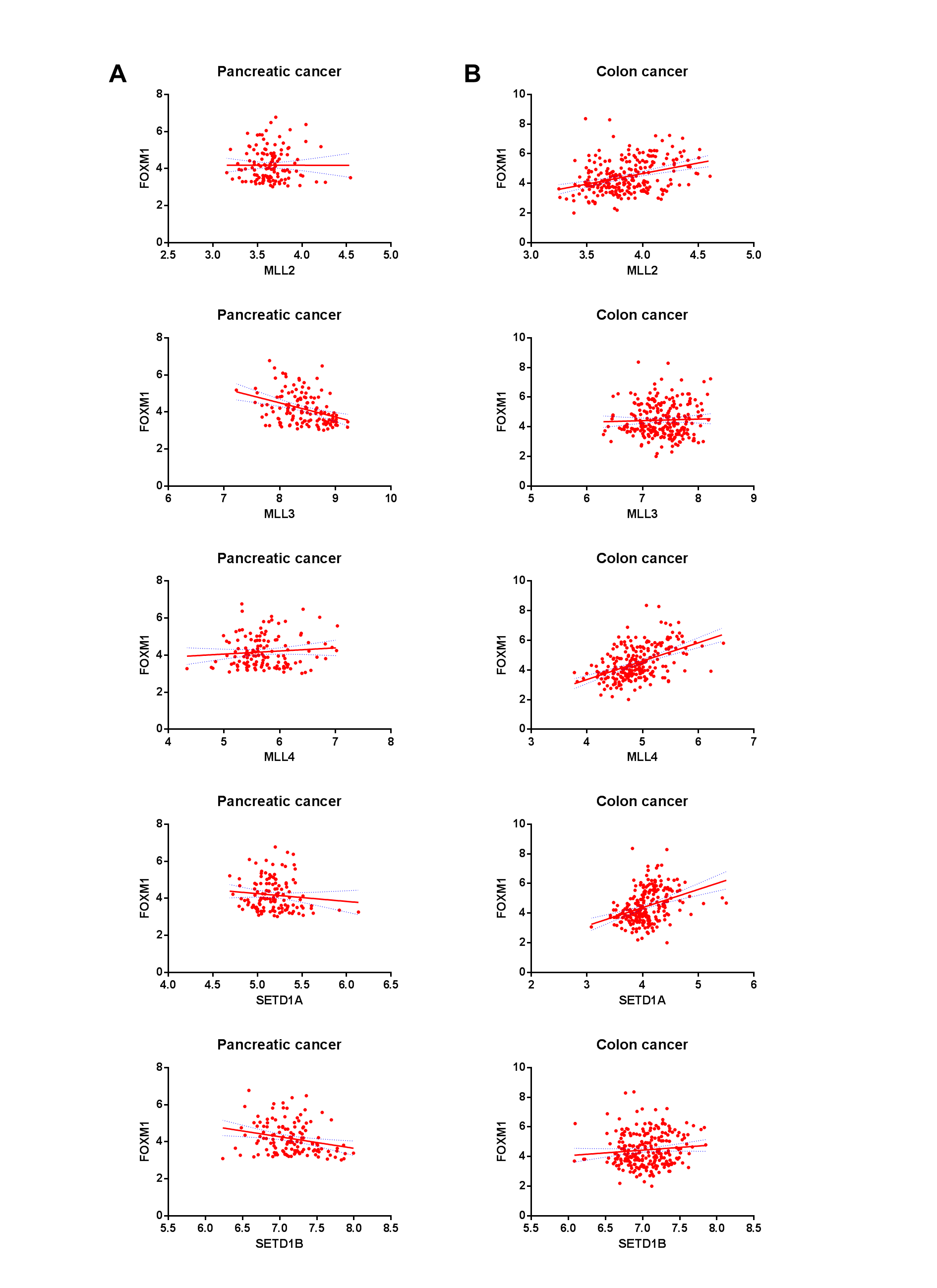

Supplement: Supplementary file 3 — Fig. S3. The correlation between the histone methyltransferase and FOXM1 in pancreatic cancer and colon cancer. [file MOL2-13-873-s003.tif]

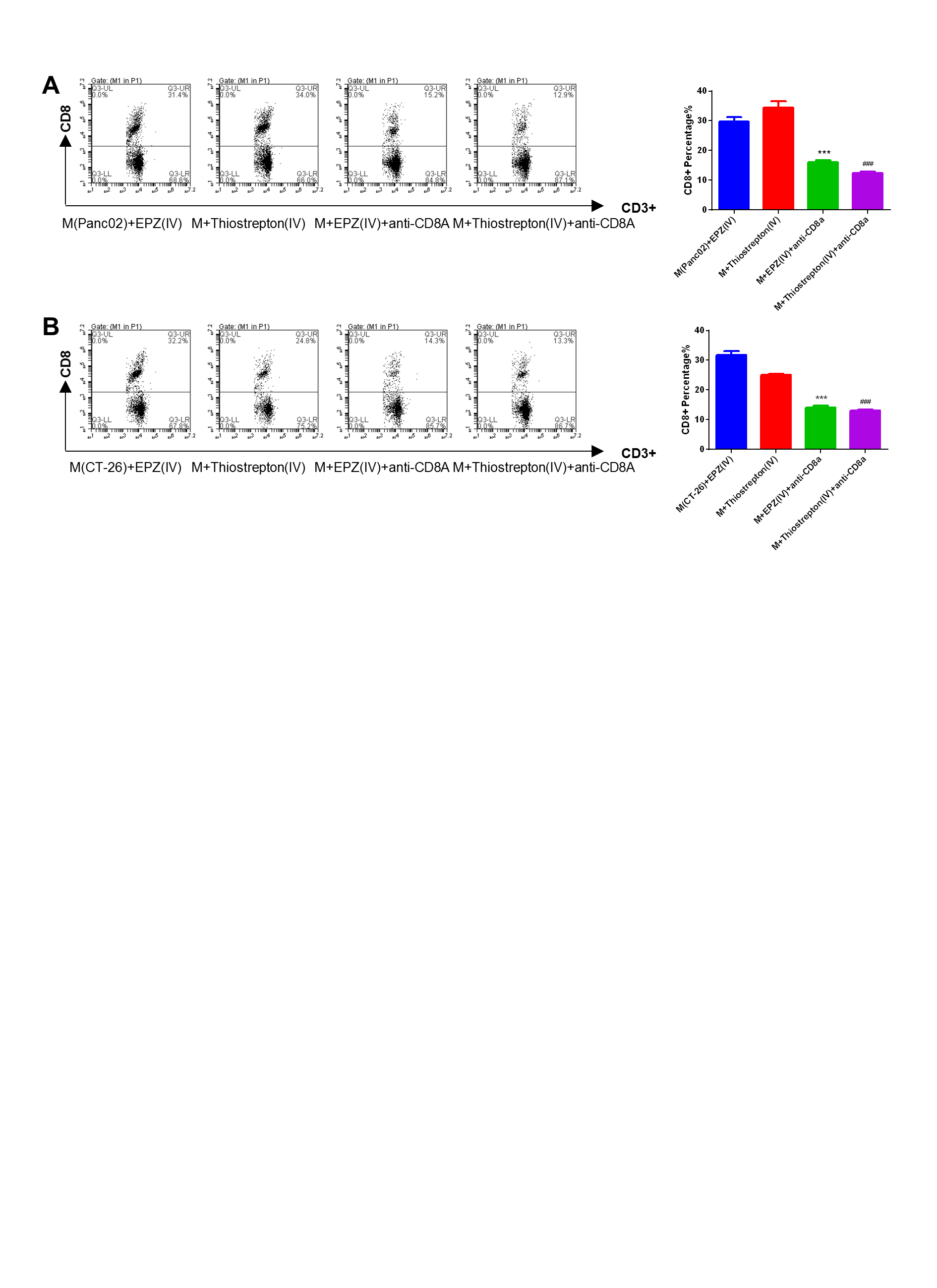

Supplement: Supplementary file 4 — Fig. S4. Anti‐CD8a inhibited CD8 + T cell population in vivo. [file MOL2-13-873-s004.tif]

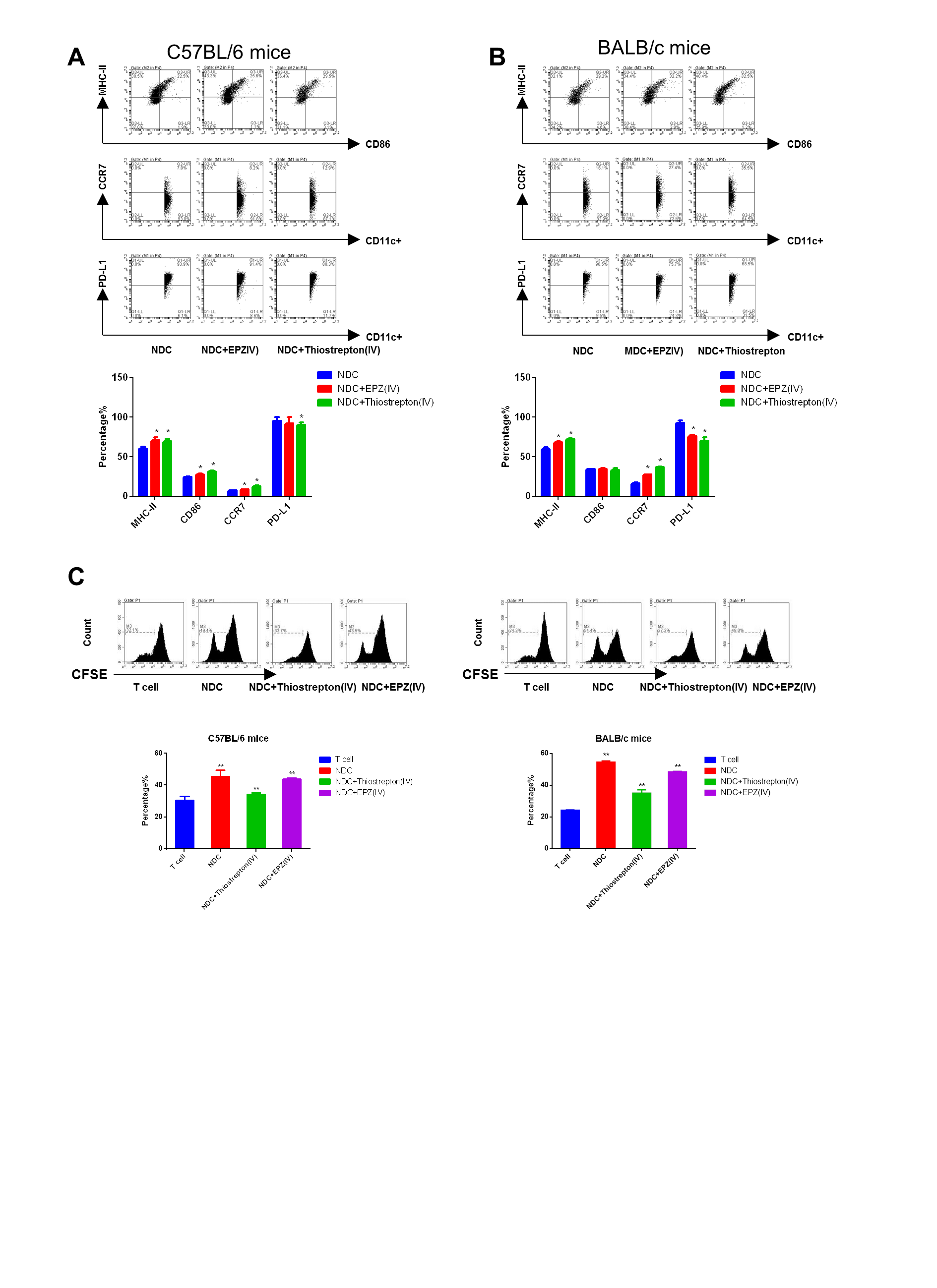

Supplement: Supplementary file 5 — Fig. S5. EPZ and Thiostrepton improved normal BMDCs maturation. [file MOL2-13-873-s005.tif]

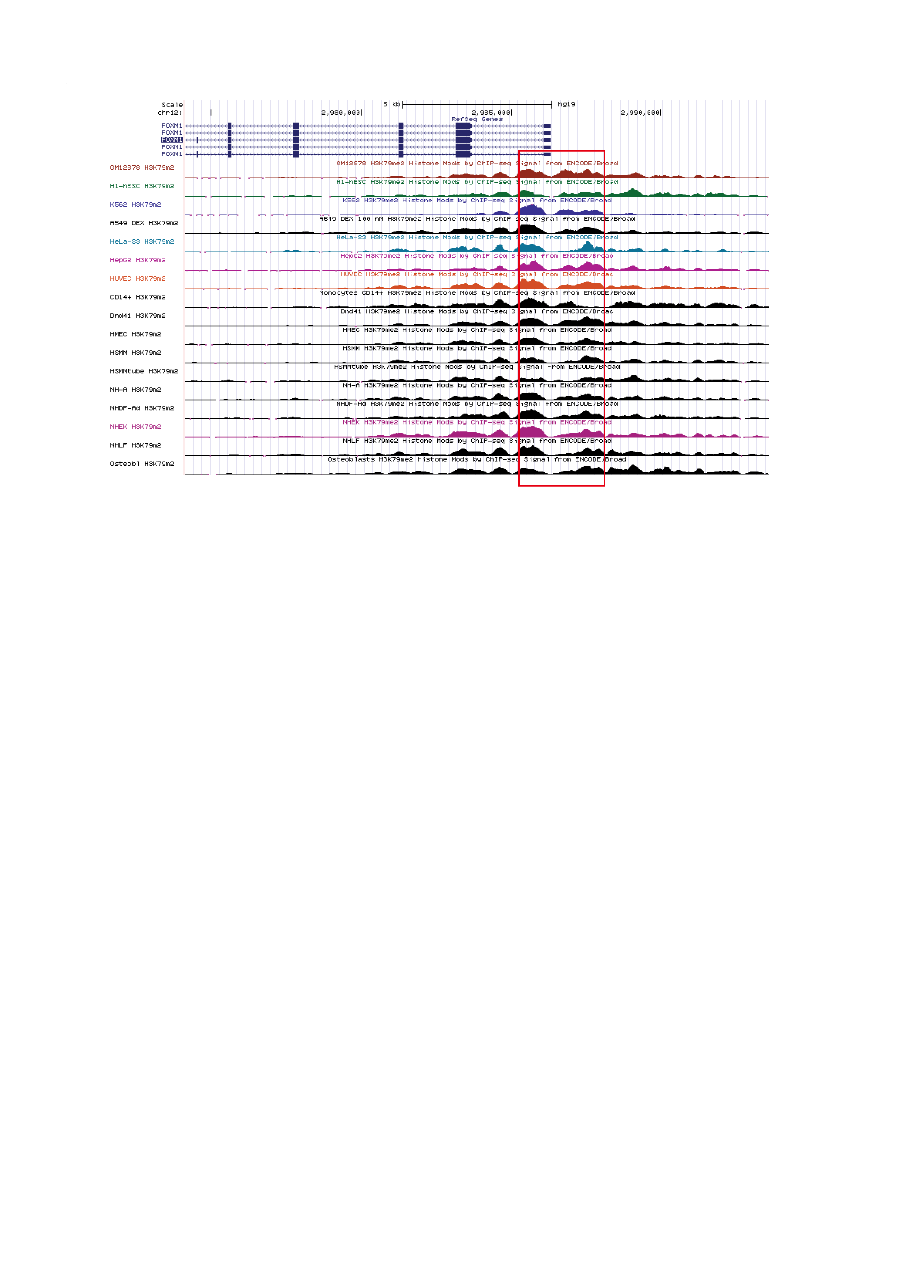

Supplement: Supplementary file 6 — Fig. S6. H3K79me2 modification tracks in human. [file MOL2-13-873-s006.tif]
