# Supplementary material for: Epigenetically modulated FOXM1 suppresses dendritic cell maturation in pancreatic cancer and colon cancer
Source: Mol Oncol. 2019 Feb 15;13(4):873–93. doi: 10.1002/1878-0261.12443 (PMC6441919; doi:10.1002/1878-0261.12443)
Supplement: Supplementary file 7 — Table S1. The sequences of qRT‐PCR primers. [file MOL2-13-873-s007.docx]

**Table S1. The sequences of qRT-PCR primers**

| **Genes** | **Forward (5’-----3’)** | **Reverse (5’-----3’)** |
| --- | --- | --- |
| β-actin(homo) | TCAGAAGGATTCCTATGTGGGCGA | TTTCTCCATGTCGTCCCAGTTGGT |
| FOXM1(homo) | TGCCCAGCAGTCTCTTACCT | CTACCCACCTTCTGGCAGTC |
| β-actin(mus) | GATTACTGCTCTGGCTCCTAGC | GACTCATCGTACTCCTGCTTGC |
| Foxm1(mus) | GCCATGATACAGTTTGCCATC | AGAGAAAGGTTGTGACGAATAGAG |
| Ccna2(mus) | GTCCTTGCTTTTGACTTGGC | ACGGGTCAGCATCTATCAAAC |
| Ccnb1(mus) | CTGACCCAAACCTCTGTAGTG | CCTGTATTAGCCAGTCAATGAGG |
| Wnt5a(mus) | CAACTGGCAGGACTTTCTCAA | CCTTCTCCAATGTACTGCATGTG |
| Foxm1-chip(mus) | GCTGGCAATGTAGACCTGG | TTGAAGAGTGAGCAGAGTGTG |
| Wnt5a-chip(mus) | CCCAGACCCTGCTTTCTC | CGCCATGCTTCTCTCATT |
