# Supplementary material for: Epigenetically modulated FOXM1 suppresses dendritic cell maturation in pancreatic cancer and colon cancer
Source: Mol Oncol. 2019 Feb 15;13(4):873–93. doi: 10.1002/1878-0261.12443 (PMC6441919; doi:10.1002/1878-0261.12443)
Supplement: Supplementary file 8 [file MOL2-13-873-s008.docx]

**Figure S1: Gating strategy used to define BMDCs and T cells populations.**

(A) BMDCs population were first gated on FSC versus SSC plot, after gating on live singlet cells (a), then data were analyzed under the CD11C^+^ gate (b), the FMO controls were used to accurately define the negative and positive cell populations (the black lines) after properly compensation by the single staining tube (c-f). (B) Splenocytes were gated before (a) and after (e) cellular permeabilization using FSC versus SSC plot. The CD4^+^ and CD8^+^ T cell populations were analyzed under CD3^+^ gate (b), the CD25^+^ and Foxp3^+^ regulatory T cell populations were analyzed under CD4^+^ gate (f)，and the FMO controls were used to accurately define the negative and positive cell populations (the black lines) after properly compensation by the single staining tube (c-d, g-h).

**Figure S2: The expression of FOXM1 at basic line.**

(A) The *FOXM1* mRNA expression of bone marrow cells, on day zero, no culture, at basic line were determined by qRT-PCR. (B) The FOXM1protein expression of bone marrow cells, on day zero, no culture, at basic line were determined by immunofluorescent staining. Scale bars, 50µm.

**Figure S3: The correlation between the** [**histone methyltransferase**](http://dict.cnki.net/dict_result.aspx?searchword=%e7%bb%84%e8%9b%8b%e7%99%bd%e7%94%b2%e5%9f%ba%e8%bd%ac%e7%a7%bb%e9%85%b6&tjType=sentence&style=&t=histone+methyltransferase) **and FOXM1 in pancreatic cancer and colon cancer.**

(A) The correlation between the [histone methyltransferase](http://dict.cnki.net/dict_result.aspx?searchword=%e7%bb%84%e8%9b%8b%e7%99%bd%e7%94%b2%e5%9f%ba%e8%bd%ac%e7%a7%bb%e9%85%b6&tjType=sentence&style=&t=histone+methyltransferase) (MLL2, MLL3, MLL4, SET1A, SET1B for H3K4me3)and FOXM1 in pancreatic cancer. (B) The correlation between the [histone methyltransferase](http://dict.cnki.net/dict_result.aspx?searchword=%e7%bb%84%e8%9b%8b%e7%99%bd%e7%94%b2%e5%9f%ba%e8%bd%ac%e7%a7%bb%e9%85%b6&tjType=sentence&style=&t=histone+methyltransferase) and FOXM1 in colon cancer.

**Figure S4: Anti-CD8a inhibited CD8^+^ T cell population *in vivo*.**

(A)-(B) The Expressions of CD8a, CD4 on gated CD3^+^ T cells, from spleen were assessed by FACS. The amounts of CD3^+^ cells set at 10000. A is from C57BL/6J, B is from BABL/c mice. Data were shown as means ± SD from at least 3 independent experiments. ^***^P<0.001 compared with the M (Panc02) +EPZ (IV), ^##^P<0.01 compared with the M + Thiostrepton (IV).

**FigureS5: EPZ and Thiostrepton improved normal BMDCs maturation.**

(A)-(B) The expression levels of CD86, MHC-II, CCR7 and PD-L1 on gated CD11c^+^ cells in normal BMDCs surfaces were all assessed by FACS. Data were shown as means ± SD from at least 3 independent experiments. ^*^P<0.05, compared with the NDC. (C) The proliferation of T cells co-cultured with normal BMDCs treated with Thiostrepton and EPZ or not was detected by CFSE assay.

**Figure S6: H3K79me2 modification tracks in human .**

Represented active H3K79me2 ChIP-Seq profiles of enrichment at FOXM1 promoter in in various human tissues and cell lines.
